# A candidate gene association analysis identifies SNPs potentially involved in drought tolerance in European beech (*Fagus sylvatica* L.)

**DOI:** 10.1038/s41598-021-81594-w

**Published:** 2021-01-27

**Authors:** Laura Cuervo-Alarcon, Matthias Arend, Markus Müller, Christoph Sperisen, Reiner Finkeldey, Konstantin V. Krutovsky

**Affiliations:** 1 Department of Forest Genetics and Forest Tree Breeding, Georg-August University of Göttingen, Büsgenweg 2, 37077 Göttingen, Germany; 2 Physiological Plant Ecology, University of Basel, Schönbeinstrasse 6, 4056 Basel, Switzerland; 3 Swiss Federal Institute for Forest, Snow and Landscape Research WSL, Zürcherstrasse 111, 8903 Birmensdorf, Switzerland; 4 University of Kassel, Mönchebergstrasse 19, 34109 Kassel, Germany; 5 Vavilov Institute of General Genetics, Russian Academy of Sciences, 3 Gubkina Str., Moscow, Russia 119333; 6 Laboratory of Foresty Genomics, Genome Research and Education Center, Siberian Federal University, 50a/2 Akademgorodok, Krasnoyarsk, Russia 660036; 7 Department of Ecosystem Science and Management, Texas A&M University, 2138 TAMU, College Station, TX 77843-2138 USA

**Keywords:** Genetics, Genetic association study, Genotype, Plant genetics, Population genetics

## Abstract

Studies of genetic variation underlying traits related to drought tolerance in forest trees are of great importance for understanding their adaptive potential under a climate change scenario. In this study, using a candidate gene approach, associations between SNPs and drought related traits were assessed in saplings of European beech (*Fagus sylvatica* L.) representing trees growing along steep precipitation gradients. The saplings were subjected to experimentally controlled drought treatments. Response of the saplings was assessed by the evaluation of stem diameter growth (SDG) and the chlorophyll fluorescence parameters F_V_/F_M_, PI_abs_, and PI_tot_. The evaluation showed that saplings from xeric sites were less affected by the drought treatment. Five SNPs (7.14%) in three candidate genes were significantly associated with the evaluated traits; saplings with particular genotypes at these SNPs showed better performance under the drought treatment. The SNPs were located in the cytosolic class I small heat-shock protein, CTR/DRE binding transcription factor, and isocitrate dehydrogenase genes and explained 5.8–13.4% of the phenotypic variance. These findings provide insight into the genetic basis of traits related to drought tolerance in European beech and could support the development of forest conservation management strategies under future climatic conditions.

## Introduction

European beech (*Fagus sylvatica* L.) is one of the ecologically and economically most important forest tree species in Europe. Its natural distribution depends mainly on temperature and moisture availability^1^, and although it is widely distributed over the continent, it is predominant in Central Europe with relatively mild climatic conditions^1,2^. The climate change scenarios predict increase in frequency, duration, and intensity of summer droughts as a consequence of rising temperature and diminishing precipitation^3,4^ that could negatively affect survival of this species^5–7^. Indeed, some studies indicate that severe drought periods might be harmful for European beech, affecting growth, leaf conductance, photosynthetic activity, stem hydraulic conductivity, fine root vitality, and nutrient uptake^8–14^. Consequently, under a current climate change scenario, it is believed that European beech could lose its habitat to more drought tolerant species, such as *Quercus petraea* and *Pinus sylvestris*^9,15^.

Under drought stress conditions plants respond by adjusting various physiological and morphological traits^16^. Stomata are closed to minimize water loss, but it reduces CO_2_ intake and, as a consequence, can result in reduction of photosynthetic activity^17,18^. Photosynthetic activity can be further reduced by inhibition of ATP synthesis^19^ and by changes in photosystem II (PSII) photochemistry that lead to reduction in electron transport^20,21^. Furthermore, changes in biomass allocation can also occur under drought stress leading to a change in the root/shoot ratio with a higher investment in root biomass to maximize soil water uptake^22^. In addition to minimizing water loss and maximizing water uptake, plants can also regulate cell osmotic potential to tolerate dehydration. It depends on such compounds as amino acids, hydrophilic proteins, and carbohydrates, which allow not only maintenance of water absorption and cell turgor under drought conditions, but also protect cell membrane and metabolic mechanisms. This osmotic adjustment is especially important in roots, allowing their growth under drought^16,17^.

It is well-known from the current literature that varying soil characteristics, such as texture, acidity, and nutrient availability, may greatly affect tree growth and its allocation to leaves, stem, and root organs^23–27^. Some studies have shown distinct soil effects on the seasonal timing of leaf development and the metabolism of carbon and nitrogen^28–30^. All this may in turn affect the responsiveness of trees to drought through interacting effects on mechanisms controlling tree water balances and mediating physiological and molecular stress tolerance. Indeed, previous studies have shown such interacting effects of drought and soil on annual shoot growth, root development, nitrogen and carbon metabolism, leaf water status, synthesis of anti-oxidative compounds and osmotic stress defense ^26,31–34^. This demonstrates that the soil may play a significant role in the function of trees under water limited conditions and thus deserves further attention in experimental studies elaborating the drought tolerance of trees.

Common garden experiments and provenance trials have shown that local adaptation is common in forest trees being reflected in differences in adaptive phenotypic traits that match local environmental conditions^1–5^. Indeed, morphological and physiological data suggest that despite its presumed susceptibility to drought, populations of European beech from dry sites are more tolerant to drought than populations from wet sites^35–38^. For example, by monitoring physiological parameters such as leaf water potential and carbon isotopic discrimination during a 3 year period including the year 2003, one of the driest years in Europe, it was found that beech trees growing in a xeric Mediterranean environment did not demonstrate substantial signs of drought stress compared to beech trees in Central Europe^39^. These findings were further supported by dendroecological data^40,41^ and drought experiments with seedlings. When exposed to drought, not only water potential, transpiration rates and growth are less affected in seedlings from xeric sites, but also the root/shoot ratio is higher, which facilitates access to soil water^34,42,43^. Additionally, studies based on genetic markers such as AFLPs and SNPs have shown differences in genetic variation between beech populations growing in environments with different moisture availability^44,45^. Thus, although these results suggest that there is local adaptation to drought in European beech, little is still known about the genetic variation underlying drought tolerance in this species.

The genetic variation underlying many traits in forest trees is likely to be polygenic, controlled by a large number of loci with small and moderate effects^46–48^. A common approach for the identification of genetic polymorphisms underlying adaptive traits is to test for associations between phenotypes and genetic variation^49,50^. Association studies can be classified as Genome-Wide Association Studies (GWAS) and candidate gene-based association studies^51^. GWAS allow searching for genetic associations with adaptive variation throughout the genome; however, it is still rather expensive and requires numerous genome-wide markers that often are noncoding or have unknown gene function^52,53^. In contrast, candidate gene-based association studies use genetic variation in genes that are likely directly involved in genetic control of adaptive traits of interest^49,51,54^. Several studies using this approach in plants have been able to successfully identify associations between SNPs in candidate genes and phenotypic traits such as disease resistance^55^, cold-hardiness^56^, bud and flower phenology^57,58^, and wood property traits^59^.

Among different genetic markers, single nucleotide polymorphisms (SNPs) are the preferred choice of molecular genetic markers for association studies, since they are the most common polymorphisms in the genome. They are found in both genic and intergenic regions and can represent mutations that might lead to changes in phenotype^60–62^. Recently, the development of SNP markers in candidate genes that are potentially involved in traits related to climate has been reported for European beech^63–65^. Not only associations between some of these SNPs and important traits, such as bud burst, bud set, growth rate, chlorophyll fluorescence, and transpiration have been detected^66,67^, but also their association with different geographic and environmental variables, such as elevation, temperature, precipitation, and aridity^45,68,69^, suggesting their relevance for the study of genetic variation important for adaptation to climate change in European beech.

Precipitation gradients might reflect differences in water availability that could promote local adaptation. Therefore, their study represents a good environmental approach to search for genetic variation underlying drought tolerance related traits. In this study, saplings of European beech from populations occurring along steep precipitation gradients in Switzerland were collected. A previous study showed signatures of natural selection in these saplings^45^ indicating the importance of further exploring genetic variation that could be involved in drought tolerance in these populations. In this study, the saplings from twelve beech populations along steep precipitation gradients in the upper Rhine and Rhône valleys in Switzerland were selected and subjected to simulated summer drought conditions. The drought simulation experiment with the selected saplings was conducted on two contrasting types of natural forest soil, with acidic or calcareous characteristic, respectively, to account for potentially interfering soil effects. Photosynthesis and growth are critical processes affected by drought^18^, therefore, they were used to evaluate the performance of the saplings under such conditions. Photosynthetic performance was measured using the following three parameters: maximum quantum efficiency of PSII (F_V_/F_M_), performance index of PSII on absorption basis (PI_abs_), and total performance index of PSII (PI_tot_). Growth was evaluated as increment in stem diameter growth (SDG). The association between these traits and 70 SNPs in 23 candidate genes potentially involved in drought response was tested.

## Results

### Drought stress intensity

After employing the two summer drought treatments in 2013 and 2014, volumetric soil water content decreased gradually to about 0.10 m^3^ m^−3^ on acidic soil and to 0.07 and 0.11 m^3^ m^−3^ on calcareous soil in 2013 and 2014, respectively. Volumetric soil water content in regularly irrigated control plots ranged in both soil types always above 0.15 m^3^ m^−3^ (Supplementary Table S1). Predawn leaf water potentials measured across a subset of saplings on acidic soil (mesic population Mastrils and xeric population Saxon) showed that the trees actually suffered from the applied drought treatments (Supplementary Table S2). Towards the end of each treatment period, average predawn leaf water potentials dropped to rather low values approaching − 1.40 MPa (SE ± 0.09) in August 2013 and − 1.81 MPa (SE ± 0.07) in August 2014. Regularly irrigated control trees maintained predawn leaf water potential at − 0.21 MPa (SE ± 0.03) in 2013 and − 0.20 MPa (SE ± 0.02) in 2014 (Supplementary Table S2).

### Statistical differences in phenotypic traits

**Figure 1:**
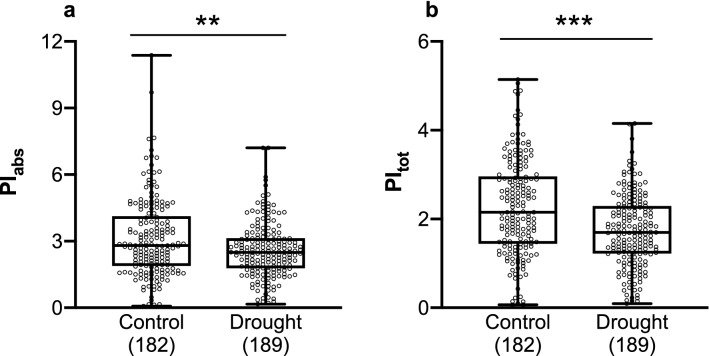
Chlorophyll fluorescence parameters PI_abs_ (**a**) and PI_tot_ (**b**) measured in the control and drought treated saplings. **P < 0.01; ***P < 0.001. Box plots present median, upper, and lower quartiles; whiskers show minimum and maximum values. Sample size is presented in parentheses.

Mixed-effects model analysis did not reveal significant differences among populations for the chlorophyll fluorescence traits (Supplementary Tables S2–S5). In contrast, the effect of chamber was significant for F_V_/F_M_ (*F*_(14,345)_ = 2.69, *P* = 0.001), PI_abs_ (*F*_(14,355)_ = 7.79, *P* < 0.001) and PI_tot_ (*F*_(14,156)_ = 6.81, *P* < 0.001). Likewise, the effect of treatment was significant for PI_abs_ (*F*_(1,355)_ = 15.24, *P* < 0.001) and PI_tot_ (*F*_(1,156)_ = 33.69, *P* < 0.001): saplings under drought treatment demonstrated lower PI_abs_ and PI_tot_ than control saplings (Fig. 1): mean differences between drought and control saplings were significant for PI_abs_ (mean difference = 0.539, *t*_(369)_ = 3.42, *P* < 0.01) and for PI_tot_ (mean difference = 0.5313, *t*_(369)_ = 5.36, *P* < 0.001). No significant differences were observed between populations under the drought treatment for the chlorophyll fluorescence parameters.

Mixed-effects model analysis for SDG showed that differences among saplings growing in different chambers were not significant (Supplementary Tables S6–S8), but, in contrast, they were significant regarding treatment (SDG 2013: *F*_(1,32)_ = 18.88, *P* < 0.001; SDG 2014: *F*_(1,23)_ = 97.36, *P* < 0.001; SDG 2013–2014: *F*_(1,27)_ = 58.46, *P* < 0.001); significant and close to significant regarding soil (SDG 2014: *F*_(1,11)_ = 18.57, *P* < 0.001; SDG 2013–2014: *F*_(1,11)_ = 4.11, *P* = 0.067); and significant or close to significant regarding population (SDG 2013: *Z* = 1.58, *P* = 0.057; SDG 2014: *Z* = 1.57, *P* = 0.059; SDG 2013–2014: *Z* = 1.83, *P* < 0.05).

**Figure 2:**
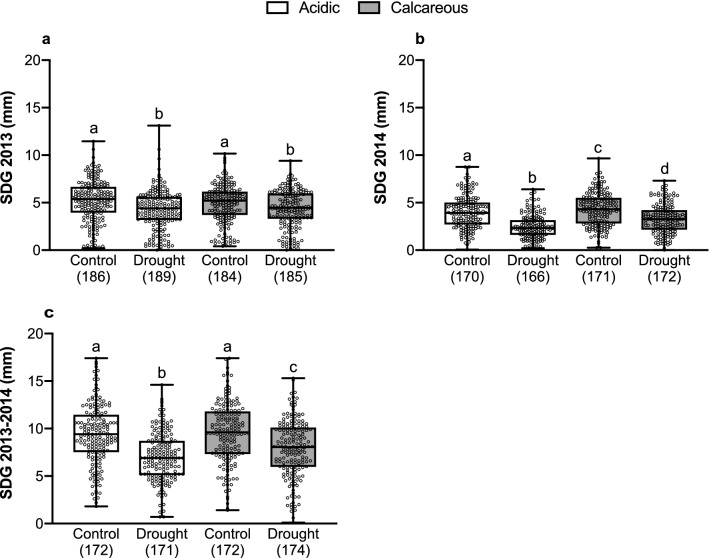
Stem diameter growth (SDG) measured in 2013 (**a**), 2014 (**b**), and 2013–2014 (**c**) in the control and drought treated saplings from acidic and calcareous soils. Different letters (a, b, c, and d) above whiskers indicate significant differences (*P* < 0.05). Box plots present median, upper, and lower quartiles; whiskers show minimum and maximum values. Sample size is presented in parentheses.

Saplings under drought demonstrated lower SDG than control saplings (Fig. 2). On acidic soil, mean differences between saplings under drought and control treatments were significant for SDG 2013 (mean difference = 0.905, *t*_(373)_ = 4.15, *P* < 0.001), SDG 2014 (mean difference = 1.525, *t*_(334)_ = 9.43, *P* < 0.001) and SDG 2013–2014 (mean difference = 2.356, *t*_(341)_ = 7.50, *P* < 0.001). On calcareous soil, mean differences were also significant for SDG 2013 (mean difference = 0.585, *t*_(365)_ = 2.84, *P* < 0.01), SDG 2014 (mean difference = 1.045, *t*_(341)_ = 5.93, *P* < 0.001), and SDG 2013–2014 (mean difference = 1.634, *t*_(344)_ = 4.91, *P* < 0.001).

Soil also affected SDG (Fig. 2). Saplings on calcareous soil had higher SDG than saplings on acidic soil showing significant mean differences for SDG 2014 under control (mean difference = 0.332, *t*_(339)_ = 1.78, *P* = 0.05) and drought (mean difference = 0.812, *t*_(336)_ = 5.42, *P* < 0.001) conditions, and for SDG 2013–2014 under drought (mean difference = 0.835, *t*_(343)_ = 2.75, *P* < 0.01). Indeed, the interaction between treatment and soil was close to significant for SDG 2014 (*F*_(1,19)_ = 3.49, *P* = 0.067) , and the results show that the effect of soil on SDG is more obvious under drought conditions, especially for SDG 2014 and SDG 2013–2014 (Fig. 2b,c).

ANOVA analysis showed that there were significant differences in SGD among populations under the drought treatment (SDG 2013: *F*_(11,694)_ = 4.217, *P* < 0.001; SDG 2014: *F*_(11,631)_ = 4.677, *P* < 0.001; SDG 2013–2014: *F*_(11,641)_ = 6.561, *P* < 0.001). The *t*-test for equality of means revealed that there were significant mean differences between Saxon, one of the most xeric populations, and other four populations with higher precipitation. In all cases, saplings from Saxon showed higher SDG (Table 1).

**Table 1:** Significant differences for the SDG traits between populations under drought treatment based on the *t* test for equality of means.

Trait	Soil	Population 1	Mean ± SE	Population 2	Mean ± SE	Mean difference (1–2)	*P*-value
			Population 1		Population 2		
SDG 2013	Acidic	Saxon	5.85 ± 0.49	Felsberg	3.28 ± 0.48	2.567	0.015
	Calcareous	Saxon	5.77 ± 0.49	Felsberg	3.17 ± 0.51	2.605	0.019
		Saxon	5.77 ± 0.49	Sargans	3.13 ± 0.51	2.639	0.016
SDG 2014	Calcareous	Saxon	4.79 ± 0.38	Felsberg	2.56 ± 0.42	2.229	0.005
		Saxon	4.79 ± 0.38	Sargans	2.01 ± 0.40	2.780	0.000
SDG 2013–2014	Acidic	Saxon	9.84 ± 0.74	Malans	6.00 ± 0.74	3.843	0.022
		Saxon	9.84 ± 0.74	Sargans	5.49 ± 0.74	4.356	0.003
		Saxon	9.84 ± 0.74	Martigny	5.93 ± 0.72	3.918	0.013
	Calcareous	Saxon	10.56 ± 0.72	Felsberg	5.97 ± 0.79	4.593	0.001
		Saxon	10.56 ± 0.72	Sargans	5.81 ± 0.77	4.748	0.000

### Association analysis

Seventy SNPs were used for the association analyses because six of the 76 initially selected SNPs turned out to be monomorphic (*APX1_2, PhyB, 50_320, 52_1_249, 92_166,* and *110_1_111*). The comparison between GLM and MLM showed that both models produced similar results having similar distribution of *P*-values in the quantile–quantile plots (Supplementary Figs S1 and S2). However, the MLM performed a little better, as demonstrated by more similar distributions of the expected and observed *P*-values (Supplementary Figs. S1 and S2). In addition, although relatedness between pairs of individuals was weak (the *r*_QG_ coefficients varied mostly between 0.0 and 0.1), saplings collected underneath the same adult tree were more related having a higher proportion of the *r*_QG_ coefficients above 0.1 (Supplementary Fig. S3). This indicates that accounting for relatedness was important for reducing false positives in the association analysis. Thus, the results of the MLM for the association analysis are also presented.

The association analysis using MLM revealed that among the 70 SNPs analyzed, five SNPs (7.14%) showed significant association with the PI_abs_, PI_tot_, and SDG traits, while two SNPs (*PP2C_315* and *7_520*) showed a trend toward significant association with the F_V_/F_M_ index (Table 2). The phenotypic variation explained by the SNPs (*R*^2^) with significant association was relatively high, between 5.8% and 13.4% (Table 2), and three of them were associated with more than one trait (*50_39*, *IDH_1*, and *IDH_4*). They were located in coding regions: one of them representing a non-synonymous substitution (*50_39*), and the rest, synonymous substitutions (*110_1_293*, *IDH_1*, *IDH_4*, and *50_232*).

**Table 2:** SNPs showing significant or close to significant association with chlorophyll fluorescence traits (FV/FM, PI_abs_, and PI_tot_) and stem diameter growth (SDG) in different experimental conditions and for the pooled individuals (all saplings).

Trait	SNP	Drought						Control (acidic soil)			All saplings			SNP type	Gene
		Acidic soil			Calcareous soil			*P*	P*	*R*^2^, %	*P*	P*	*R*^2^, %		
		*P*	P*	*R*^2^, %	*P*	P*	*R*^2^, %								
F_V_/F_M_	*PP2C_315*	0.00^**+**^	0.15^**+**^	5.1	ND	ND	ND	0.14	0.94	2.9	0.00^**+**^	0.12^**+**^	4.1	Non-synonymous	*Protein phosphatase 2C*
	*7_520*	0.00	0.19	7.3	ND	ND	ND	0.89	0.99	0.0	0.00^**+**^	0.12^**+**^	4.1	Non-coding	*Xyloglucan endotransglucosylase hydrolase 23*
PI_abs_	*110_1_293*	0.07	0.79	3.8	ND	ND	ND	**0.00**	**0.02**	13.4	**0.00**	**0.03**	5.8	Synonymous	*Cytosolic class I small heat-shock protein*
PI_tot_	*50_39*	**0.00**	0.11^**+**^	10.6	ND	ND	ND	0.50	0.99	1.3	0.03	0.78	2.4	Non-synonymous	*CTR/DRE binding factor*
SDG 2013	*50_39*	**0.00**	0.14^**+**^	8.5	0.96	0.99	0.0	0.09	0.91	3.6	0.47	0.99	0.3	Non-synonymous	*CTR/DRE binding factor*
SDG 2014	*IDH_1*	0.85	0.99	0.4	**0.00**	**0.07**	10.2	0.85	0.99	0.4	0.01	0.72	1.4	Synonymous	*Isocitrate dehydrogenase*
	*IDH_4*	0.49	0.99	1.3	**0.00**	**0.07**	10.8	0.40	0.99	1.6	0.01	0.72	1.5	Synonymous	*Isocitrate dehydrogenase*
SDG 2013–2014	*IDH_1*	0.99	0.99	0.0	**0.00**	0.11^**+**^	8.8	0.99	0.99	0.0	0.05	0.87	1.1	Synonymous	*Isocitrate dehydrogenase*
	*IDH_4*	0.59	0.99	1.0	**0.00**	**0.07**	9.7	0.46	0.99	1.4	0.04	0.87	1.1	Synonymous	*Isocitrate dehydrogenase*
	*50_39*	0.00^**+**^	0.14^**+**^	8.2	0.92	0.98	0.1	0.90	0.99	0.3	0.88	0.99	0.1	Non-synonymous	*CTR/DRE binding factor*
	*50_232*	**0.00**	0.14^**+**^	8.4	0.86	0.98	0.2	0.70	0.99	0.8	0.98	0.99	0.0	Synonymous	*CTR/DRE binding factor*

P*—adjusted *P* value; *R*^2^—percent of phenotypic variation explained by the SNP; ND—not determined; ^+^—close to significance. In bold are *P* values that were still significant after applying the Bonferroni correction (*P* ≤ 0.0014) or after adjusting for a FDR < 0.1.

**Figure 3:**
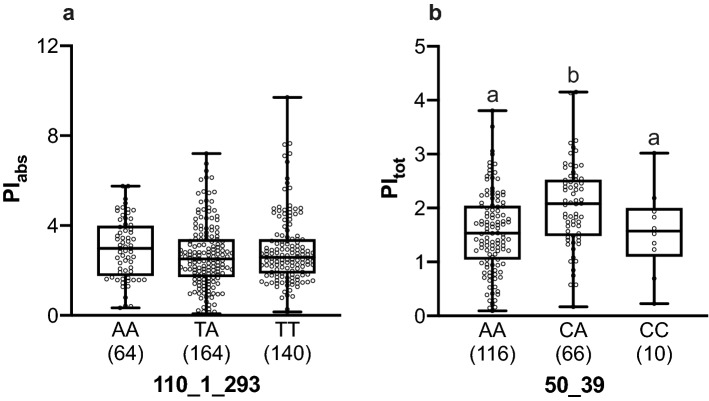
Chlorophyll fluorescence parameters PI_abs_ (**a**) and PI_tot_ (**b**) measured in homozygote (AA and TT) and heterozygote (TA) genotypes of the A/T SNP *110_1_293* (**a**) and in homozygote (AA and CC) and heterozygote (CA) genotypes of the A/C SNP *50_39* (**b**) that were significantly associated with these parameters under control/acidic (**a**) and drought/acidic (**b**) soil conditions, respectively. Different letters (a and b) above whiskers indicate significant differences (*P* < 0.05). Box plots present median, upper and lower quartiles; whiskers show minimum and maximum values. Sample size is presented in parentheses.

**Figure 4:**
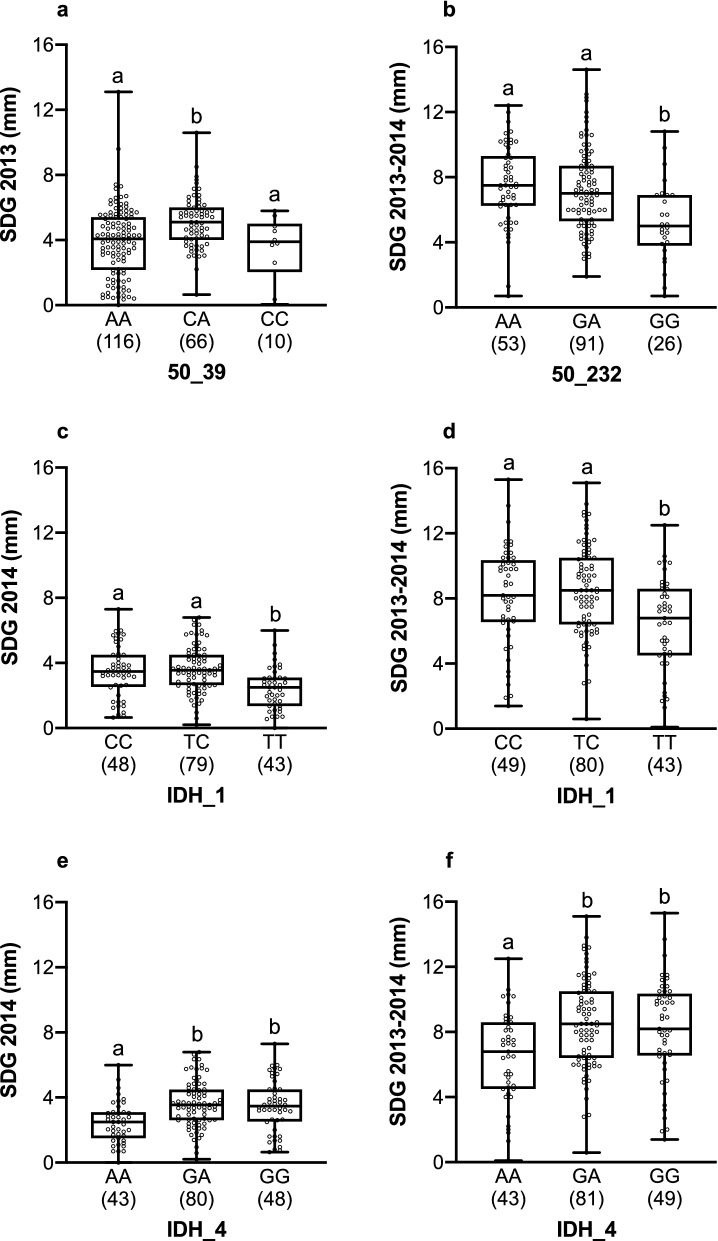
Stem diameter growth (SDG) measured in homozygote and heterozygote genotypes of four SNPs (*50_39*, *50_232*, *IDH_1*, and *IDH_4*) that were significantly associated with SDG under drought/acidic (**a** and **b**) and drought/calcareous (**c**, **d**, **e**, and **f**) soil conditions. Different letters (a and b) above whiskers indicate significant differences (*P* < 0.05). Box plots present median, upper and lower quartiles; whiskers show minimum and maximum values. Sample size is presented in parentheses.

Interestingly, each significant association was found only in particular conditions, i.e., the association between SNP and trait was significant only in specific experimental and soil conditions (Table 2). In all cases the significant associations were observed under drought conditions, except the SNP *110_1_293* in the *Cytosolic class I small heat-shock protein* gene, which was associated with the PI_abs_ index for chlorophyll fluorescence under control rather than drought conditions. Homozygous individuals TT for this SNP showed a mean PI_abs_ by 9% higher than homozygotes AA and heterozygotes TA, even though the differences were not significant (Fig. 3a). The PI_tot_ index was significantly associated with the SNP *50_39* in the *CTR/DRE binding factor* gene under drought/acidic soil conditions. Heterozygotes CA at this SNP showed significantly higher PI_tot_ (by 31% on average) than homozygotes AA (*P* < 0.001, Fig. 3b). In contrast, no significant differences were found between heterozygotes CA and homozygotes CC, although heterozygotes CA showed higher PI_tot_ (Fig. 3b). The SNP *50_39* also showed significant association with SDG 2013 under drought/acidic soil conditions; heterozygous saplings showed significantly higher SDG (by 38% on average) than AA (*P* < 0.001) and CC (*P* < 0.05) saplings (Fig. 4a). Another SNP from the same gene, *50_232,* was associated with SDG 2013–2014 also under drought/acidic soil conditions. Homozygotes GG for this SNP had significantly lower SDG (by 28.3% on average) than homozygotes AA (*P* < 0.001, Fig. 4b) and heterozygotes GA (*P* < 0.01, Fig. 4b).

In addition, two SNPs from the *IDH* gene, *IDH_1* and *IDH_4* SNPs, were associated with SDG 2014 and SDG 2013–2014 under drought/calcareous soil conditions. For *IDH_1*, homozygotes TT had significantly lower SDG compared to homozygotes CC in 2014 (*P* < 0.01) and 2013–2014 (*P* < 0.01), and compared to heterozygotes TC in 2014 (*P* < 0.001) and 2013–2014 (*P* < 0.001, Fig. 4c, d), with a mean SDG by 23.4% lower for the whole period 2013–2014. Likewise, for the *IDH_4* SNP, homozygous saplings AA had significantly lower SDG when compared to homozygotes GG in 2014 (*P* < 0.01) and 2013–2014 (*P* < 0.01), and also compared to heterozygotes GA in 2014 (*P* < 0.001) and 2013–2014 (*P* < 0.001, Fig. 4e, f), with a mean SDG by 22.6% lower for the whole period SDG 2013–2014.

Spearmans’ correlation analysis showed that correlation between individual heterozygosity and the traits was not significant in most comparisons, except F_V_/F_M_ and SDG that showed significant association with individual heterozygosity under drought but not under control conditions (Supplementary Table S9).

## Discussion

Photosynthesis is a key physiological process in plants. It depends on environmental factors such as light, temperature, and water availability, therefore, its evaluation can provide information on the response of plants to different environmental stresses, indicating whether the plant is being affected. Since chlorophyll fluorescence is correlated with photosynthetic rates, the evaluation of chlorophyll fluorescence is a widely used approach to assess the impact of stress on photosynthesis^70,71^.

Three different parameters based on chlorophyll fluorescence, which provide information on distinct phases of the photochemical process^72^ were assessed in our study. It was found that F_V_/F_M_ was not affected by the drought treatment, since no significant differences were found between saplings under control vs. drought conditions. It has been reported that F_V_/F_M_ has low responsiveness to drought stress and is not a good indicator to evaluate drought stress tolerance^73–78^. In contrast, PI_abs_ and PI_tot_ were better indicators of tolerance to drought stress. Significant differences in PI_abs_ and PI_tot_ were found between saplings under control vs. drought treatments (Fig. 1). Saplings under drought treatment demonstrated lower values of PI_abs_ and PI_tot_ indicating that the biochemical performance of PSII was negatively affected by drought conditions. Similar negative impact on PSII measured by chlorophyll fluorescence has been found in other studies on European beech saplings under drought conditions^73,76,78^.

Although there were no significant differences among populations in chlorophyll fluorescence traits, there is evidence suggesting that saplings from the studied xeric sites recover faster after drought^76^. Other studies also suggest that beech trees from dry habitats are less affected by drought based on the assessment of such parameters as carbon isotopic composition, transpiration rate, and water potential^39,42^. Further exploration by including more individuals and populations from precipitation gradients could help to find actual differences in chlorophyll fluorescence related traits.

Growth is another important trait to evaluate plant responses to different stresses, as it is also affected by environmental conditions. It was observed that under the drought treatment, SDG of all saplings was negatively affected (Fig. 2). Other studies have also reported reductions in diameter increment, ring width, and height growth in beech under a soil water deficit^29,34,79,80^. This reduction in SDG could be explained not only by a decrease in carbon assimilation under drought conditions^81,82^ and a prioritization of carbon storage over growth^29^, but also by changes in biomass allocation priorities resulting in an increase in the root/shoot ratio to facilitate access to soil water^8,43^. Interestingly, in 2014, a higher reduction in SDG was observed in saplings subjected to the drought treatment compared to 2013 (Fig. 2). In fact, European beech has a delayed growth response to drought that results in a more marked reduction the next year after the drought occurs^83^, which could explain the higher reduction in SDG observed in 2014.

Additionally, the type of soil also influenced SDG. Indeed, neighboring populations with very similar climate but growing on different soil types can show marked differences in growth^84^. It is known that although European beech is able to grow on different soil types, humid calcareous soils are the most favorable for its optimum growth^85^. In fact, saplings growing on calcareous soil showed higher SDG than saplings growing on acidic soil, especially in 2014 and the overall SDG 2013–2014 (Fig. 2). This effect of soil type on SDG is expected since soil characteristics influence water availability and nutrient uptake, and consequently affect growth^9,29,86^. Furthermore, it was found that the effect of the drought treatment was enhanced by the soil type: saplings growing on acidic soil and under drought conditions showed the lowest SDG (Fig. 2b,c). Similar results were reported by Thiel et al.^34^, who found that under drought conditions a sandy soil with lower water storage capacity and lower nutrient availability had a stronger negative impact on the performance of beech compared to a loamy soil.

Interestingly, although the drought treatment had a negative impact on SDG, comparison among saplings under the drought treatment showed that Saxon, one of the most xeric populations, showed higher SDG than saplings from populations with higher precipitation regimes, indicating that saplings from Saxon were less affected by drought (Table 1). These findings are in agreement with other studies reporting that populations of beech from xeric sites are less sensitive to drought^34,40,43^, indicating adaptation to such conditions.

Potentially, adaptive genetic variation can be detected by associating variation of adaptive phenotypic traits with allelic variation^50^. Nevertheless, population structure developed due to selectively neutral genetic processes such as genetic drift and relatedness can cause false associations^53^. Thus, it is necessary to account for them in association analyses. In the present study, the inclusion of both population structure and relatedness inferred using the selectively neutral microsatellite markers reduced the chance of false associations. This was demonstrated by a similar distribution between the expected and the observed *P*-values in the quantile–quantile plots (Supplementary Figs S1 and S2). Furthermore, although there was weak genetic relatedness between pairs of saplings, the saplings collected underneath the same adult tree were more related (Supplementary Fig. S3) confirming that accounting for relatedness in addition to accounting for population structure was also important for the reduction of false positive associations. Thus, the SNPs deviating significantly from the expected *P*-values are likely to be truly associated with the studied phenotypic traits.

In total, five SNPs (7.14% of all SNPs) demonstrated significant association with the studied traits (Table 2). The phenotypic variation explained by these SNPs was relatively high (5.8–13.4%), compared to the results of other studies reporting values between 2.1 and 6.9% in beech and other species^59,66,87,88^. Several reasons could explain these findings. First, controlled experimental conditions could heighten differences in response to drought and, thus, increase the proportion of phenotypic variation explained by the SNPs. Second, candidate genes that participate in stress response were specifically selected for this study, increasing the chances of finding SNPs that could have an effect on the phenotypic traits. However, it cannot be ruled out that sample size could have an effect on the percentage of phenotypic variation explained by the SNPs. Studies with small population sizes could be affected by the Beavis effect that refers to the statistical effect of overestimation of marker-trait association due to small sample sizes in QTL or association studies^89^. It is estimated that a sample size of 1000 individuals would be needed to avoid such overestimation^48^. In our study, a total of 755 saplings and only 187–193 saplings per experimental conditions (treatment/soil) were used (Supplementary Table S10). Thus, an overestimation of *R*^2^due to low sample size cannot be ruled out. Furthermore, the presence of linkage disequilibrium (LD) could also inflate *R*^2^ of an individual marker, since it could jointly contribute to the trait together with other markers^48^. Among the significant SNPs, LD was found between pairs of SNPs in the same gene: between *IDH_1* and *IDH_4*, and between *50_39* and *50_232*^45^. Thus, an inflation of *R*^2^due to LD also cannot be ruled out. In addition, it is difficult to identify the likely causal SNP when it is in LD with other SNPs that are also significantly associated with a trait.

Noteworthy, significant associations between the SNPs and the phenotypic traits were found only in particular experimental and soil conditions (Table 2). Indeed, the adaptive value of genetic variation and its influence on fitness are highly dependent on the context^90^. Thus, under control/acidic soil conditions, the *110_1_293* SNP in the *Cytosolic class I small heat-shock protein* gene was significantly associated with the chlorophyll fluorescence index PI_abs_. Nevertheless, the two alleles at this SNP seem to have low differences in their phenotypic effect, since the differences in the performance of the different genotypes were not significant (Fig. 3a). In contrast, the *50_39* SNP in the *CTR/DRE binding factor* gene demonstrated association under drought/acidic soil conditions with both PI_tot_ and SDG 2013; apparently, heterozygotes had a better performance under such conditions (Figs. 3b and 4a), indicating overdominance. Under the same conditions, another SNP from the same gene, *50_232,* showed association with SDG 2013–2014 (Table 2). Allele A at this SNP was strongly associated with faster SDG under such conditions (Fig. 4b) indicating a dominant mode of action.

Similarly, the *IDH_1* and *IDH_4* SNPs in the *Isocitrate dehydrogenase* gene also showed a dominant mode of action: alleles C in *IDH_1* and G in *IDH_4* were associated with faster SDG under drought/calcareous conditions (Fig. 4). Noteworthy, *IDH_1* and *IDH_4* were also found to be associated with environmental variables in the original sites such as temperature, precipitation, and humidity^45^ indicating that they are very likely to be involved in adaptation of the studied populations. Indeed, previous studies in other tree species using isoenzymes also identified the *IDH* gene to be significantly involved in tolerance to environmental stresses such as drought and temperature^91,92^. Kim et al.^93^ showed increase of the *IDH* gene expression under drought conditions in maize.

All the SNPs showing significant association with the studied traits were located in coding regions, but only *50_39* represented a non-synonymous substitution, while *110_1_293*, *IDH_1*, *IDH_4*, and *50_232*—synonymous substitutions. Non-synonymous SNPs are considered the most likely target of natural selection because they cause changes in protein sequence, and thus, might directly affect the phenotype^61^. Thus, the synonymous SNPs showing significant association in the present study could be tightly linked to non-synonymous SNPs that were not genotyped. However, synonymous SNPs could also be under direct selection and cause changes in phenotypic traits, since they can affect mRNA splicing, mRNA stability, and translation kinetics^94–96^ or affect translation and protein synthesis due to tRNA bias^97,98^. This highlights the importance of considering not only non-synonymous SNPs but also synonymous and non-coding SNPs, since they could have a key role in gene expression and post-transcriptional regulation and, thus, influence important adaptive phenotypic traits^47,62,99,100^.

The *7_520* and *PP2C_315* SNPs showed close to significant association with the chlorophyll fluorescence index F_V_/F_M_. One of the requirements to evaluate association is that the phenotypic trait should be variable enough^53^. It was not the case for F_V_/F_M_ and that could limit the detection of associations for this trait. However, low sample sizes could also limit the detection of associations. Since complex traits in forest trees are usually controlled by many loci with small to moderate phenotypic effect, large sample sizes are required to detect their effect^53,101,102^. Indeed, it is estimated that population sizes in the range of hundreds or even thousands would be needed^48^. Thus, further exploration with larger sample size and a better coverage of European beech distribution area would be needed not only to confirm association of the *7_520* and *PP2C_315* SNPs with F_V_/F_M_, but also to discover new associations important for adaptation to drought^103^.

It has been also estimated that the number of causative loci underlying polygenic traits in plants could be in the order of several hundred^48^. Although candidate gene-based association studies are less demanding regarding the number of markers required because they provide a direct link to genes that supposedly affect the trait of interest, they might miss important genetic variation in other genes not included in the study that could be relevant, but that have not been identified yet^51,53,54^. With the development of recent technologies such as Genotype by Sequencing (GBS) the discovery of SNPs throughout the genome of non-model species is now more feasible^104,105^, making possible the implementation of GWAS. This, together with the recent publication of a reference genome of European beech^106^, will help to get more insight into the genetic basis of drought tolerance in this species.

Heterozygosity has usually been considered as advantageous for fitness. For example, highly heterozygous individuals in crops and domesticated animals can express heterosis—better vigor and yields^107^. Heterozygosity was positively associated with growth, stress tolerance, and survival in different pine species^91,107,108^. However, heterozygosity did not always strongly correlate with fitness^109,110^. Indeed, our results show that only heterozygous individuals at *50_39* SNP in the *CTR/DRE binding factor gene* showed better performance over homozygotes (Figs. 3b and 4a), and, in most of the cases, no significant correlation between total multi-locus individual heterozygosity and the traits was found (Supplementary Table S9). Only F_V_/F_M_ and SDG 2013 showed significant association with individual heterozygosity under drought conditions. However, the magnitude of the correlation coefficients (0.09–0.17) indicated a relatively weak correlation. Therefore, the better performance of some saplings cannot be attributed to the general level of heterozygosity in the studied SNPs. Further exploration including additional SNPs that provide a better coverage of the genome could lead to a better estimation of the whole-genome heterozygosity and shed light on its association with the performance of the saplings.

In addition to genetic variation, epigenetic variation could also be responsible for differences in phenotypic traits. Epigenetic marks could also explain some of the phenotypic variation that is usually attributed to genetic variation. Thus, if it has an effect on adaptive phenotypic traits and is stable enough for selection to act, epigenetic variation could provide long lasting selective advantage and be important for local adaptation^46,111^. During stress conditions, epigenetic changes play an important role leading to acclimation responses via phenotypic plasticity^101,111^. Moreover, some epigenetic changes can be very stable and inherited from one generation to the next, and thus, facilitate adaptation in important traits such as drought tolerance^112,113^. Even though our results showed that some genotypes confer a better performance under drought conditions, which suggest genetic adaptation, it cannot be ruled out that acclimation, and thus, phenotypic plasticity, could have also played a significant role in the performance of the saplings. Indeed, the tested SNPs could represent adaptive genetic variation and also be involved in acclimation, since their genes participate in stress response. Although it is difficult to disentangle the relative contributions that phenotypic plasticity and genetic variation have on the phenotype^114^, future research should take into consideration both genetic and epigenetic variation, as both contribute to adaptation to environmental changing conditions.

## Material and methods

### Plant material and experimental design

Twelve beech populations along steep precipitation gradients in the upper Rhine and Rhône valleys in Switzerland were selected (Table 3). From each population, 16–31 adult trees about 50 m apart from each other were selected. Underneath each tree, 2–4 saplings approximately 20 cm tall and 3–5 years old were excavated, for a total of 60–64 saplings per population. In total, 755 saplings were transplanted in spring 2011 in a randomized design to the model ecosystem facility MODOEK of the Swiss Federal Institute for Forest, Snow and Landscape Research (WSL) in Birmensdorf, Switzerland to be subjected to simulated summer drought conditions.

**Table 3:** Geographic and climate data for the period 1981–2010^a^ for beech populations used in the study.

Valley	Population	Number of saplings	Latitude	Longitude	Elevation^30^, m.a.s.l	Mean annual	
						precipitation, *mm*	temperature, °C
Rhine	Felsberg	62	46.854	9.487	650–800	849	10.0
	Chur	63	46.863	9.548	700–800	849	10.0
	Malans	64	46.986	9.570	600–700	1114	10.1
	Mastrils	62	46.970	9.543	550–650	1114	10.1
	Sargans	63	47.056	9.444	650–750	1334	10.1
	Mels	60	47.053	9.411	650–750	1334	10.1
Rhone	Ardon	63	46.220	7.246	750–850	603	10.1
	Chamoson	64	46.212	7.214	750–850	603	10.1
	Saxon	64	46.146	7.191	700–800	603	10.1
	Martigny	64	46.104	7.108	500–700	855	10.1
	Collombey	63	46.272	6.933	550–650	1012	9.8
	Ollon	63	46.303	6.997	600–700	1012	9.8

^a^Taken from the METEO SWISS stations located near the populations (distance ≤ 10 km).

The MODOEK facility is composed of 16 chambers equipped with an automated irrigation system for the control of water supply. Each chamber has a sliding roof and is split below ground in two concrete-walled lysimeters with a depth of 150 cm, each containing acidic (Haplic Alisol) or calcareous (Fluvisol) forest soil of similar texture with a pH of 4.0 and 6.9, respectively^26^. In a randomized design, two saplings per population were transplanted on each type of soil in each chamber. Two growing seasons (2011 and 2012) were used for saplings acclimatization. To exclude natural precipitation and control water irrigation, the sliding roofs of the chambers were closed from May to October. During that period, the saplings were irrigated every 2 or 3 days with 50 l of water per m^−2^ resembling ambient rainfall composition^26^. During hot periods, the amount and frequency of irrigation were incremented to keep soil water content above 0.15 m^3^ m^−3^ at 10 cm soil depth and counterbalance high evapotranspiration.

Drought conditions were imposed in eight of the chambers in 2013 and 2014 by excluding water irrigation from May to August. In particularly hot days, short irrigation pulses were applied to avoid too fast soil drying and irreversible damage of the saplings. The remaining eight chambers were used as control by not applying drought conditions and maintaining irrigation every 2 or 3 days as described above. A summary on the number of saplings from each population under each treatment (control and drought), type of soil (acidic and calcareous), and chamber is provided in Supplementary Table S11.

Water relations of both soil and saplings was monitored to assess soil drought and physiological stress intensities. In each lysimeter, volumetric soil water content was measured at 10 cm soil depth using PC-controlled moisture probes and 5TM soil moisture and temperature sensor (Decagon Devices, Inc., Pullman, WA, USA). Predawn leaf water potential was measured across a subset of 58 saplings on acidic soil (mesic population Mastrils and xeric population Saxon) using a Scholander pressure chamber M 600 (Mosler Tech Support, Berlin, Germany).

### Evaluation of phenotypic traits

In July 2014, chlorophyll fluorescence was assessed as previously described^78^ to evaluate the leaf physiological stress response of the transplanted saplings growing on acidic soil. In short, fast fluorescence kinetics were measured in the dark-adapted leaves between 11:00 and 12:00 using a portable plant efficiency analyzer (Pocket PEA, Hansatech Instruments Ltd., Norfolk, UK). The increment in fluorescence was recorded after a saturating light pulse of 3500 μmol quanta m^2^/s of red light (650 nm). Based on the obtained fluorescence kinetics, different parameters describing the stages of the photochemical process with regard to energy absorption, trapping and electron transport are obtained providing information on stress-induced impairments of the photosystem II. We selected the following three fluorescence related parameters: (1) the maximum quantum efficiency of PSII (F_V_/F_M_), which provides information on the fraction of the total energy flux trapped by PSII reaction centers; (2) the performance index of PSII on absorption basis (PI_abs_), which provides information on energy conservation from photon trapping to the reduction of intersystem electron acceptors; and (3) the total performance index of PSII (PI_tot_), which provides information on energy conservation from photon trapping to the reduction of final electron acceptors in PSI^72,115^.

In addition, the SDG was measured in all saplings as an indicator of morphological response to the simulated drought conditions. SDG was measured 5 cm above ground in March 2013, October 2013 and September 2014. Increment in SDG was calculated as the difference in stem diameter between March-October 2013 (SDG 2013), October 2013-September 2014 (SDG 2014) and March 2013-September 2014 (SDG 2013–2014).

### DNA extraction

Leaves from the saplings were collected, dehydrated with silica gel and stored at room temperature until DNA extraction. DNA extraction was performed with the DNeasy 96 Plant Kit (Qiagen, Hilden, Germany). To examine the amount and quality of the extracted DNA, electrophoresis in 1% agarose gel using 1X TAE as running buffer was performed. DNA was stained and visualized with Roti-Safe GelStain (Roth, Karlsruhe, Germany) under UV illumination, and compared with a Lambda DNA size ladder (Roche, Mannheim, Germany).

### Microsatellite amplification

To establish the genetic relatedness among pairs of individuals, saplings were genotyped at 13 highly polymorphic microsatellite markers. Three of them were EST-linked markers originally developed for *Quercus robur*—*GOT066*, *FIR065* and *FIR004*^116^. The rest of the markers were random genomic microsatellites developed for *F. crenata* (*sfc0018, sfc0161, sfc1063*)^117^ and *F. sylvatica* (*FS3-04, msf11, csolfagus_06, csolfagus_19, Fagsyl_002929, Fagsyl_003994*)^118–121^. The markers were pooled in four multiplexes for the PCR amplification: all EST markers were included in the first multiplex; all *sfc* markers in the second multiplex; *FS3-04* and *msf11* markers in the third multiplex; and *csolfagus* and *Fagsyl* markers in the fourth multiplex. Primers for the *GOT066*, *FIR065*, *sfc0018*, *sfc1143, FS3-04,* and *Fagsyl_002929* markers were labeled with 6-hexachlorofluorescein (HEX) fluorescent dye, whereas primers for the *FIR004, sfc0161*, *sfc1063, mfs11*, *csolfagus_06*, *csolfagus_19*, and *Fagsyl_003994* markers were labeled with 6-carboxyfluorescein (FAM) fluorescent dye. The PCR amplifications were carried out in a final volume of 15 μL containing about 10 ng of genomic DNA, 1X reaction buffer (0.8 M Tris–HCl pH 9.0, 0.2 M (NH_4_)_2_SO_4_, 0.2% *w/v* Tween-20; Solis BioDyne, Tartu, Estonia), 2.5 mM MgCl_2_, 0.2 mM of each dNTP, 1 unit of *Taq* DNA polymerase (HOT FIREPol DNA Polymerase, Solis BioDyne, Tartu, Estonia), and 0.3 μM of each primer. The PCR protocol contained an initial denaturation at 95 °C for 15 min, 30 cycles of 1 min denaturation at 94 °C, 30 s annealing at 47 °C (first multiplex) or at 55 °C (second, third, and fourth multiplexes), and 1 min extension at 72 °C. The final extension step was performed at 72 °C for 20 min followed by the hold at 16 °C. The PCR fragments were separated in an ABI 3130xl Genetic Analyzer (Applied Biosystems, Foster City, USA), and the fragment sizes were scored using the GeneMapper 4.1 software (Applied Biosystems, Foster City, USA) based on the internal size standard GS 500 ROX (Applied Biosystems, Foster City, USA).

### Selection of candidate genes and SNPs

To assess genetic variation possibly underlying the evaluated traits, candidate genes were selected as described in Cuervo-Alarcon et al.^45^. In brief, based on previous studies on SNP discovery in European beech^63–65^, candidate genes that very likely participate in stress response according to the UniProt (www.uniprot.org) and the Arabidopsis Information Resource (TAIR) (www.arabidopsis.org) databases^122,123^ were selected. SNPs for genotyping in those genes were chosen based on their identification as haplotype tag SNPs by the software htSNPer 1.0^124^ or because they already showed signs of natural selection in previous studies^66,68^. For primer design, sequences surrounding the SNPs were sent to LGC Genomics Ltd. where SNP genotyping was performed using the PCR-based KASP genotyping assay (Hoddesdon, UK). In total, 70 polymorphic SNPs in 23 candidate genes were genotyped in the saplings and represented 19 non-synonymous, 26 synonymous and 25 non-coding SNPs (Supplementary Table S12).

### Statistical analysis of phenotypic traits

Normality and homogeneity of variances for each trait was tested using Shapiro–Wilk test and Levene’s test, respectively, using the IBM SPSS statistics software (https://www.ibm.com/analytics/spss-statistics-software). F_V_/F_M_ was not normally distributed for most of the groups (Supplementary Table S13); in contrast, data for the rest of the traits was normally distributed either for the majority or for all the groups (Supplementary Tables S14–S18). As mixed-effects models are robust to departures from normality, they were used to test for statistically significant differences in both chlorophyll fluorescence and SDG traits using the Minitab Statistical Software (https://www.minitab.com).

For the chlorophyll fluorescence traits F_V_/F_M_, PI_abs_, and PI_tot_, the effects of population, treatment, and chamber were tested for significant differences, while soil was not accounted for because chlorophyll fluorescence was assessed only in saplings growing on acidic soil. For the mixed-effects model, population was considered as a random factor, whereas treatment and chamber were considered fixed factors. In addition, a *t*-test for equality of means was performed to compare saplings under control vs. drought treatment, and also one way-ANOVA was carried out to test for differences among populations under drought treatment. Both *t*-test and ANOVA were performed using the IBM SPSS statistics software (https://www.ibm.com/analytics/spss-statistics-software).

For the SDG traits, effects of population, treatment, soil and chamber were tested for significant differences. For the mixed-effects model, population was considered a random factor, whereas treatment, chamber, and soil were considered fixed factors. Besides, *t*-tests for equality of means to compare saplings under control vs. drought treatment, saplings on acidic vs. calcareous soil and populations under drought treatment were carried out using the IBM SPSS statistics software (https://www.ibm.com/analytics/spss-statistics-software).

### Association analysis

Associations between SNPs and the phenotypic traits were tested using the TASSEL 5.0 software^125^. For the analyses, individuals were grouped according to the experimental conditions: treatment and treatment/soil. Besides, the analysis for all saplings together regardless of experimental conditions was also performed. In this case, phenotypic data were normalized by dividing the data by the mean of each experimental condition. Association analyses were performed using the general linear model (GLM) and the mixed linear model (MLM). The GLM took into account population structure that potentially could be developed due to selectively neutral genetic processes such as genetic drift as a confounding factor (*Q*), where the *Q-*matrix was calculated using the tentatively selectively neutral microsatellite markers and the STRUCTURE 2.3.4 software^126^ as described in Cuervo-Alarcon et al.^45^. The MLM took into account both *Q* and kinship (*K*) as confounding factors. The *K*-matrix was calculated also based on the microsatellite genotypes as the pairwise relatedness coefficient *r*_QG_ estimated according to Queller and Goodnight^127^ using the GenAlEx software^128,129^; negative values of *r*_QG_ were set to zero. Two different methods were used to account for multiple testing in GLM and MLM analyses: Bonferroni correction using *P*-value cut-off equaled 0.00143 and adjustment of *P*-values using the false discovery rate^130^ (FDR equal to 0.1) implemented in the R function “p.adjust”^131^. Only SNPs with a minimum allele frequency (MAF) higher than 0.05 were used for the association analysis.

Furthermore, relationships between heterozygosity and the measured traits were evaluated. Individual heterozygosity (proportion of loci that are heterozygous across an individual) was calculated using the GenAlEx 6.5 software^128,129^. Spearman’s correlation analyses between individual heterozygosity and the traits were performed using the R 3.3.1 software^131^.

## Supplementary Information

Supplementary Information.

## Data Availability

The data on candidate genes, polymorphic SNPs, respective nucleotide sequences, their PCR primers and the NCBI GeneBank and the EMBL nucleotide sequence database accession numbers used in this study are publicly available and provided in Table S12 and references cited there.

## References

[1] Fang J, Lechowicz MJ (2006). Climatic limits for the present distribution of beech (*Fagus* L.) species in the world. J. Biogeogr..

[2] Ellenberg H (1988). Vegetation ecology of Central Europe.

[3] Trenberth K (2011). Changes in precipitation with climate change. Clim. Res..

[4] Kovats RS, Barros VR (2014). Europe. Climate Change 2014: Impacts, Adaptation, and Vulnerability. Part B: Regional Aspects Contribution of Working Group II to the Fifth Assessment Report of the Intergovernmental Panel on Climate Change.

[5] von Wühlisch G (2008). EUFORGEN Technical Guidelines for Genetic Conservation and Use for European Beech (*Fagus sylvatica*).

[6] Gärtner S (2008). The drought tolerance limit of *Fagus sylvatica* forest on limestone in southwestern Germany. J. Veg. Sci..

[7] Kramer K (2010). Modelling exploration of the future of European beech (*Fagus sylvatica* L.) under climate change—range, abundance, genetic diversity and adaptive response. For. Ecol. Manag..

[8] Leuschner C (2001). Drought responses at leaf, stem and fine root levels of competitive *Fagus sylvatica* L. and *Quercus petraea* (Matt.) Liebl trees in dry and wet years. For. Ecol. Manag..

[9] Geßler A (2007). Potential risks for European beech (*Fagus sylvatica* L.) in a changing climate. Trees.

[10] Piovesan G, Biondi F, Filippo AD, Alessandrini A, Maugeri M (2008). Drought-driven growth reduction in old beech (*Fagus sylvatica* L.) forests of the central Apennines. Italy. Glob. Change Biol..

[11] Köcher P, Gebauer T, Horna V, Leuschner C (2009). Leaf water status and stem xylem flux in relation to soil drought in five temperate broad-leaved tree species with contrasting water use strategies. Ann. For. Sci..

[12] Milad M, Schaich H, Bürgi M, Konold W (2011). Climate change and nature conservation in Central European forests: a review of consequences, concepts and challenges. For. Ecol. Manag..

[13] Scharnweber T (2011). Drought matters—Declining precipitation influences growth of *Fagus sylvatica* L. and *Quercus robur* L. in north-eastern Germany. For. Ecol. Manag..

[14] Packham JR, Thomas PA, Atkinson MD, Degen T (2012). Biological flora of the British Isles: *Fagus sylvatica*. J. Ecol..

[15] Friedrichs DA (2009). Species-specific climate sensitivity of tree growth in Central-West Germany. Trees.

[16] Aranda I, Aroca R (2012). Drought response in forest trees: from the species to the gene. Plant Responses to Drought Stress.

[17] Chaves MM, Maroco JP, Pereira JS (2003). Understanding plant responses to drought—from genes to the whole plant. Funct. Plant Biol..

[18] Osakabe Y, Osakabe K, Shinozaki K, Tran L-SP (2014). Response of plants to water stress. Front. Plant Sci..

[19] Lawlor DW (2002). Limitation to photosynthesis in water-stressed leaves: Stomata vs metabolism and the role of ATP. Ann. Bot..

[20] Lu C, Zhang J (1999). Effects of water stress on photosystem II photochemistry and its thermostability in wheat plants. J. Exp. Bot..

[21] Wang Z (2018). Effects of drought stress on photosynthesis and photosynthetic electron transport chain in young apple tree leaves. Biol. Open.

[22] Brunner I, Herzog C, Dawes MA, Arend M, Sperisen C (2015). How tree roots respond to drought. Front. Plant Sci..

[23] Spinnler D, Egli P, Körner C (2002). Four-year growth dynamics of beech-spruce model ecosystems under CO_2_ enrichment on two different forest soils. Trees.

[24] Högberg P, Fan H, Quist M, Binkley D, Tamm CO (2006). Tree growth and soil acidification in response to 30 years of experimental nitrogen loading on boreal forest. Glob. Change Biol..

[25] Poorter H (2012). Biomass allocation to leaves, stems and roots: meta-analyses of interspecific variation and environmental control. New Phytol..

[26] Kuster TM, Arend M, Bleuler P, Günthardt-Goerg MS, Schulin R (2013). Water regime and growth of young oak stands subjected to air-warming and drought on two different forest soils in a model ecosystem experiment. Plant Biol..

[27] Kuster TM, Arend M, Günthardt-Goerg MS, Schulin R (2013). Root growth of different oak provenances in two soils under drought stress and air warming conditions. Plant Soil.

[28] Li M-H (2013). Responses of leaf nitrogen and mobile carbohydrates in different *Quercus* species/provenances to moderate climate changes. Plant Biol..

[29] Liu J-F (2017). Effects of drought on leaf carbon source and growth of European beech are modulated by soil type. Sci. Rep..

[30] Arend M, Gessler A, Schaub M (2016). The influence of the soil on spring and autumn phenology in European beech. Tree Physiol..

[31] Kuster TM, Dobbertin M, Günthardt-Goerg MS, Schaub M, Arend M (2014). A phenological timetable of oak growth under experimental drought and air warming. PLoS ONE.

[32] Hu B (2015). Changes in the dynamics of foliar N metabolites in oak saplings by drought and air warming depend on species and soil type. PLoS ONE.

[33] Hu B, Simon J, Rennenberg H (2013). Drought and air warming affect the species-specific levels of stress-related foliar metabolites of three oak species on acidic and calcareous soil. Tree Physiol..

[34] Thiel D (2014). Different reactions of central and marginal provenances of *Fagus sylvatica* to experimental drought. Eur. J. For. Res..

[35] Železnik P (2019). Root growth dynamics of three beech (*Fagus sylvatica* L.) provenances. For. Ecol. Manag..

[36] Božič G, Kraigher H, Šijačić-Nikolić M, Milovanović J, Nonić M (2019). International European beech provenance trial Kamenski hrib/Straža in Slovenia. Forests of Southeast Europe Under a Changing Climate: Conservation of Genetic Resources.

[37] Čortan D, Nonić M, Šijačić-Nikolić M, Šijačić-Nikolić M, Milovanović J, Nonić M (2019). Phenotypic plasticity of European beech from international provenance Trial in Serbia. Forests of Southeast Europe Under a Changing Climate: Conservation of Genetic Resources.

[38] Müller M, Gailing O (2019). Abiotic genetic adaptation in the Fagaceae. Plant Biol..

[39] Fotelli MN (2009). Seasonal and interannual ecophysiological responses of beech (*Fagus sylvatica*) at its south-eastern distribution limit in Europe. For. Ecol. Manag..

[40] Weber P, Bugmann H, Pluess AR, Walthert L, Rigling A (2013). Drought response and changing mean sensitivity of European beech close to the dry distribution limit. Trees.

[41] Cavin L, Jump A (2017). Highest drought sensitivity and lowest resistance to growth suppression are found in the range core of the tree *Fagus sylvatica* L. not the equatorial range edge. Glob. Change Biol..

[42] Peuke AD, Schraml C, Hartung W, Rennenberg H (2002). Identification of drought-sensitive beech ecotypes by physiological parameters. New Phytol..

[43] Rose L, Leuschner C, Köckemann B, Buschmann H (2009). Are marginal beech (*Fagus sylvatica* L.) provenances a source for drought tolerant ecotypes?. Eur. J. For. Res..

[44] Pluess AR, Weber P (2012). Drought-adaptation potential in *Fagus sylvatica*: Linking moisture availability with genetic diversity and dendrochronology. PLoS ONE.

[45] Cuervo-Alarcon L (2018). Genetic variation and signatures of natural selection in populations of European beech (*Fagus sylvatica* L.) along precipitation gradients. Tree Genet. Genomes.

[46] Alberto FJ (2013). Potential for evolutionary responses to climate change—evidence from tree populations. Glob. Change Biol..

[47] Lind BM, Menon M, Bolte CE, Faske TM, Eckert AJ (2018). The genomics of local adaptation in trees: are we out of the woods yet?. Tree Genet. Genomes.

[48] Hall D, Hallingbäck HR, Wu HX (2016). Estimation of number and size of QTL effects in forest tree traits. Tree Genet. Genomes.

[49] Balding DJ (2006). A tutorial on statistical methods for population association studies. Nat. Rev. Genet..

[50] Anderson JT, Willis JH, Mitchell-Olds T (2011). Evolutionary genetics of plant adaptation. Trends Genet..

[51] Ingvarsson PK, Street NR (2011). Association genetics of complex traits in plants. New Phytol..

[52] Pearson TA, Manolio TA (2008). How to interpret a genome-wide association study. JAMA.

[53] Korte A, Farlow A (2013). The advantages and limitations of trait analysis with GWAS: a review. Plant Methods.

[54] Franks SJ, Hoffmann AA (2012). Genetics of climate change adaptation. Annu. Rev. Genet..

[55] Álvarez MF (2017). Identification of novel associations of candidate genes with resistance to late blight in *Solanum tuberosum* group Phureja. Front. Plant Sci..

[56] Eckert AJ (2009). Association genetics of coastal Douglas Fir (*Pseudotsuga menziesii* var. *menziesii*, Pinaceae). I. Cold-hardiness related traits. Genetics.

[57] Ingvarsson PK, Garcia MV, Luquez V, Hall D, Jansson S (2008). Nucleotide polymorphism and phenotypic associations within and around the phytochrome B2 locus in European aspen (*Populus tremula*, Salicaceae). Genetics.

[58] Ehrenreich IM (2009). Candidate gene association mapping of *Arabidopsis* flowering time. Genetics.

[59] González-Martínez SC, Wheeler NC, Ersoz E, Nelson CD, Neale DB (2007). Association Genetics in *Pinus taeda* L. I. Wood property traits. Genetics.

[60] Morin PA, Luikart G, Wayne RK, the SNP workshop group (2004). SNPs in ecology, evolution and conservation. Trends Ecol. Evol..

[61] Huq A (2016). Identification of functional SNPs in genes and their effects on plant phenotypes. J. Plant Biotechnol..

[62] Mei W, Stetter MG, Gates DJ, Stitzer MC, Ross-Ibarra J (2018). Adaptation in plant genomes: Bigger is different. Am. J. Bot..

[63] Seifert S, Vornam B, Finkeldey R (2012). A set of 17 single nucleotide polymorphism (SNP) markers for European beech (*Fagus sylvatica* L.). Conserv. Genet. Resour..

[64] Lalagüe H (2014). Nucleotide diversity and linkage disequilibrium at 58 stress response and phenology candidate genes in a European beech (*Fagus sylvatica* L.) population from southeastern France. Tree Genet. Genomes.

[65] Müller M, Seifert S, Finkeldey R (2015). Identification of SNPs in candidate genes potentially involved in bud burst in European beech (*Fagus sylvatica* L.). Silvae Genet..

[66] Müller M, Seifert S, Finkeldey R (2015). A candidate gene-based association study reveals SNPs significantly associated with bud burst in European beech (*Fagus sylvatica* L.). Tree Genet. Genomes.

[67] Krajmerová D (2017). Nucleotide polymorphisms associated with climate, phenology and physiological traits in European beech (*Fagus sylvatica* L.). New For..

[68] Csilléry K (2014). Detecting short spatial scale local adaptation and epistatic selection in climate-related candidate genes in European beech (*Fagus sylvatica*) populations. Mol. Ecol..

[69] Pluess AR (2016). Genome–environment association study suggests local adaptation to climate at the regional scale in *Fagus sylvatica*. New Phytol..

[70] Maxwell K, Johnson GN (2000). Chlorophyll fluorescence—a practical guide. J. Exp. Bot..

[71] Murchie EH, Lawson T (2013). Chlorophyll fluorescence analysis: a guide to good practice and understanding some new applications. J. Exp. Bot..

[72] Bussotti F, Gerosa G, Digrado A, Pollastrini M (2020). Selection of chlorophyll fluorescence parameters as indicators of photosynthetic efficiency in large scale plant ecological studies. Ecol. Indic..

[73] Gallé A, Feller U (2007). Changes of photosynthetic traits in beech saplings (*Fagus sylvatica*) under severe drought stress and during recovery. Physiol. Plant..

[74] Robson TM, Rodríguez-Calcerrada J, Sánchez-Gómez D, Aranda I (2009). Summer drought impedes beech seedling performance more in a sub-Mediterranean forest understory than in small gaps. Tree Physiol..

[75] Arend M, Brem A, Kuster TM, Günthardt-Goerg MS (2013). Seasonal photosynthetic responses of European oaks to drought and elevated daytime temperature. Plant Biol..

[76] Arend M, Sever K, Pflug E, Gessler A, Schaub M (2016). Seasonal photosynthetic response of European beech to severe summer drought: Limitation, recovery and post-drought stimulation. Agric. For. Meteorol..

[77] Cocozza C (2016). Variation in ecophysiological traits and drought tolerance of beech (*Fagus sylvatica* L.) seedlings from different populations. Front. Plant Sci..

[78] Pflug EE (2018). Resilient leaf physiological response of European Beech (*Fagus sylvatica* L.) to summer drought and drought release. Front. Plant Sci..

[79] Bouriaud O, Bréda N, Moguédec GL, Nepveu G (2004). Modelling variability of wood density in beech as affected by ring age, radial growth and climate. Trees.

[80] Lebourgeois F, Bréda N, Ulrich E, Granier A (2005). Climate-tree-growth relationships of European beech (*Fagus sylvatica* L.) in the French Permanent Plot Network (RENECOFOR). Trees.

[81] Priwitzer T, Kurjak D, Kmeť J, Sitková Z, Leštianska A (2014). Photosynthetic response of European beech to atmospheric and soil drought. For. J..

[82] Hagedorn F (2016). Recovery of trees from drought depends on belowground sink control. Nat. Plants.

[83] Bolte A, Czajkowski T, Kompa T (2007). The north-eastern distribution range of European beech—a review. Forestry.

[84] Müller M, Finkeldey R (2016). Genetic and adaptive trait variation in seedlings of European beech provenances from Northern Germany. Silvae Genet..

[85] Jahn G, Röhrig E, Ulrich B (1991). Temperate deciduous forests. Ecosystems of the World.

[86] Piedallu C, Gégout J-C, Perez V, Lebourgeois F (2013). Soil water balance performs better than climatic water variables in tree species distribution modelling. Glob. Ecol. Biogeogr..

[87] Hao D, Chao M, Yin Z, Yu D (2012). Genome-wide association analysis detecting significant single nucleotide polymorphisms for chlorophyll and chlorophyll fluorescence parameters in soybean (*Glycine max*) landraces. Euphytica.

[88] Porth I (2013). Genome-wide association mapping for wood characteristics in Populus identifies an array of candidate single nucleotide polymorphisms. New Phytol..

[89] Beavis W, Paterson AH (1998). QTL analyses: Power, precision, and accuracy. Molecular Dissection of Complex Traits.

[90] Delph LF, Kelly JK (2014). On the importance of balancing selection in plants. New Phytol..

[91] Mopper S, Mitton JB, Whitham TG, Cobb NS, Christensen KM (1991). Genetic differentiation and heterozygosity in pinyon pine associated with resistance to herbivory and environmental stress. Evolution.

[92] Bergmann F, Gregorius HR (1993). Ecogeographical distribution and thermostability of isocitrate dehydrogenase (IDH) alloenzymes and European silver fir (*Abies alba*). Biochem. Syst. Ecol..

[93] Kim SG (2015). Physiological and proteomic analysis of the response to drought stress in an inbred Korean maize line. Plant Omics.

[94] Chamary J, Hurst LD (2005). Evidence for selection on synonymous mutations affecting stability of mRNA secondary structure in mammals. Genome Biol..

[95] Pagani F, Raponi M, Baralle FE (2005). Synonymous mutations in CFTR exon 12 affect splicing and are not neutral in evolution. Proc. Natl. Acad. Sci. USA.

[96] Komar AA (2007). SNPs, silent but not invisible. Science.

[97] Plotkin JB, Kudla G (2011). Synonymous but not the same: the causes and consequences of codon bias. Nat. Rev. Genet..

[98] Mitra S, Ray S, Banerjee R (2016). Synonymous codons influencing gene expression in organisms. Rese Rep Biochem.

[99] Shastry BS, Komar A (2009). SNPs: impact on gene function and phenotype. Single Nucleotide Polymorphisms. Methods in Molecular Biology™ (Methods and Protocols).

[100] Barrett LW, Fletcher S, Wilton SD (2012). Regulation of eukaryotic gene expression by the untranslated gene regions and other non-coding elements. Cell. Mol. Life Sci..

[101] Aitken SN, Yeaman S, Holliday JA, Wang T, Curtis-McLane S (2008). Adaptation, migration or extirpation: climate change outcomes for tree populations. Evol. Appl..

[102] Hong EP, Park JW (2012). Sample size and statistical power calculation in genetic association studies. Genomics Inform..

[103] Carsjens C (2014). Intra-specific variations in expression of stress-related genes in beech progenies are stronger than drought-induced responses. Tree Physiol..

[104] He J (2014). Genotyping-by-sequencing (GBS), an ultimate marker-assisted selection (MAS) tool to accelerate plant breeding. Front. Plant Sci..

[105] Parchman TL, Jahner JP, Uckele KA, Galland LM, Eckert AJ (2018). RADseq approaches and applications for forest tree genetics. Tree Genet. Genomes.

[106] Mishra B (2018). A reference genome of the European beech (*Fagus sylvatica* L.). GigaScience.

[107] Mitton JB, Grant MC (1984). Associations among protein heterozygosity, growth rate, and developmental homeostasis. Annu. Rev. Ecol. Syst..

[108] Sharma K, Degen B, von Wuehlisch G, Singh NB (2007). An assessment of heterozygosity and fitness in Chir pine (*Pinus roxburghii* Sarg.) using isozymes. New For..

[109] Britten HB (1996). Meta-analyses of the association between multilocus heterozygosity and fitness. Evol. Int. J. Org. Evol..

[110] Rodríguez-Quilón I (2015). Local effects drive heterozygosity–fitness correlations in an outcrossing long-lived tree. Proc. R. Soc. B Biol. Sci..

[111] Moler ERV, Rajora OP (2019). Population epigenomics: advancing understanding of phenotypic plasticity, acclimation, adaptation and diseases. Population Genomics: Concepts, Approaches and Applications.

[112] Zhang Y-Y, Fischer M, Colot V, Bossdorf O (2013). Epigenetic variation creates potential for evolution of plant phenotypic plasticity. New Phytol..

[113] González RM, Ricardi MM, Iusem ND (2013). Epigenetic marks in an adaptive water stress-responsive gene in tomato roots under normal and drought conditions. Epigenetics.

[114] Franks SJ, Weber JJ, Aitken SN (2014). Evolutionary and plastic responses to climate change in terrestrial plant populations. Evol. Appl..

[115] Strasser RJ, Tsimilli-Michael M, Qiang S, Goltsev V (2010). Simultaneous in vivo recording of prompt and delayed fluorescence and 820-nm reflection changes during drying and after rehydration of the resurrection plant *Haberlea rhodopensis*. Biochim. Biophys. Acta BBA - Bioenerg..

[116] Durand J (2010). A fast and cost-effective approach to develop and map EST-SSR markers: oak as a case study. BMC Genomics.

[117] Asuka Y, Tani N, Tsumura Y, Tomaru N (2004). Development and characterization of microsatellite markers for *Fagus crenata* Blume. Mol. Ecol. Notes.

[118] Pastorelli R (2003). Characterization of microsatellite markers in *Fagus sylvatica* L. and Fagus orientalis Lipsky. Mol. Ecol. Notes.

[119] Vornam B, Decarli N, Gailing O (2004). Spatial distribution of genetic variation in a natural beech stand (*Fagus sylvatica* L.) based on microsatellite markers. Conserv. Genet..

[120] Lefèvre S, Wagner S, Petit RJ, De Lafontaine G (2012). Multiplexed microsatellite markers for genetic studies of beech. Mol. Ecol. Resour..

[121] Pluess AR, Määttänen K (2013). Characterization of eighteen novel microsatellite markers and multiplex PCR protocol for *Fagus sylvatica*. Conserv. Genet. Resour..

[122] Apweiler R (2004). UniProt: the universal protein knowledgebase. Nucleic Acids Res..

[123] Lamesch P (2012). The arabidopsis information resource (TAIR): improved gene annotation and new tools. Nucleic Acids Res..

[124] Ding K, Zhang J, Zhou K, Shen Y, Zhang X (2005). htSNPer1.0: software for haplotype block partition and htSNPs selection. BMC Bioinform.

[125] Bradbury PJ (2007). TASSEL: software for association mapping of complex traits in diverse samples. Bioinformatics.

[126] Pritchard JK, Stephens M, Donnelly P (2000). Inference of population structure using multilocus genotype data. Genetics.

[127] Queller DC, Goodnight KF (1989). Estimating relatedness using genetic markers. Evolution.

[128] Peakall R, Smouse PE (2012). GenAlEx 6.5: genetic analysis in Excel. Population genetic software for teaching and research—an update. Bioinformatics.

[129] Peakall R, Smouse PE (2006). GENALEX 6: genetic analysis in Excel. Population genetic software for teaching and research. Mol. Ecol. Notes.

[130] Benjamini Y, Hochberg Y (1995). Controlling the false discovery rate: a practical and powerful approach to multiple testing. J. R. Stat. Soc. Ser. B Methodol..

[131] R Core Team. R: A language and environment for statistical computing. (R Foundation for Statistical Computing, 2016).

