# Supplementary material for: A candidate gene association analysis identifies SNPs potentially involved in drought tolerance in European beech (Fagus sylvatica L.)
Source: Sci Rep. 2021 Jan 27;11:2386. doi: 10.1038/s41598-021-81594-w (PMC7840767; doi:10.1038/s41598-021-81594-w)
Supplement: Supplementary file 1 — Supplementary Information. [file 41598_2021_81594_MOESM1_ESM.docx]

**Supplementary Information**

**A candidate gene association analysis identifies SNPs potentially involved in drought tolerance in European beech (*Fagus sylvatica* L.)**

Laura Cuervo-Alarcon^1^, Matthias Arend^2^, Markus Müller^1^, Christoph Sperisen^3^, Reiner Finkeldey^4^, Konstantin V. Krutovsky^1,5,6,7*^

^1^Department of Forest Genetics and Forest Tree Breeding, Faculty of Forest Sciences and Forest Ecology, Georg-August University of Göttingen, Büsgenweg 2, 37077 Göttingen, Germany

^2^Physiological Plant Ecology, University of Basel, Schönbeinstrasse 6, 4056 Basel, Switzerland

^3^Swiss Federal Institute for Forest, Snow and Landscape Research WSL, Zürcherstrasse 111, 8903 Birmensdorf, Switzerland

^4^University of Kassel, Mönchebergstrasse 19, 34109 Kassel, Germany

^5^Vavilov Institute of General Genetics, Russian Academy of Sciences, 3 Gubkina Str., Moscow 119333, Russia

^6^Laboratory of Foresty Genomics, Genome Research and Education Center, Siberian Federal University, 50a/2 Akademgorodok, Krasnoyarsk 660036, Russia

^7^Department of Ecosystem Science and Management, Texas A&M University, 2138 TAMU, College Station, TX 77843-2138, USA

*****Corresponding author. Tel: +49 551 393 35 37; Fax: +49 551 39 83 67; e-mail: [konstantin.krutovsky@forst.uni-goettingen.de](mailto:konstantin.krutovsky@forst.uni-goettingen.de); ORCID: 0000-0002-8819-7084

**Table S1.** Volumetric soil water content (Mean ± SE, m^3^ m^-3^) measured at 10 cm soil depth in each chamber during the drought experiment (May-August).

|  |  | **2013** | |  | **2014** | |
| --- | --- | --- | --- | --- | --- | --- |
| **Treatment** | **Chamber** | **Acidic soil** | **Calcareous soil** |  | **Acidic soil** | **Calcareous soil** |
| **Control** | 1.2 | 0.25 ± 0.03 | 0.18 ± 0.02 |  | 0.22 ± 0.03 | 0.13 ± 0.02 |
|  | 1.4 | 0.21 ± 0.02 | 0.20 ± 0.03 |  | 0.22 ± 0.02 | 0.17 ± 0.04 |
|  | 2.1 | 0.17 ± 0.02 | 0.20 ± 0.02 |  | 0.24 ± 0.03 | 0.17 ± 0.04 |
|  | 2.3 | 0.23 ± 0.02 | 0.16 ± 0.07 |  | 0.23 ± 0.02 | 0.19 ± 0.01 |
|  | 3.2 | 0.21 ± 0.02 | 0.16 ± 0.02 |  | 0.18 ± 0.03 | 0.15 ± 0.02 |
|  | 3.3 | 0.24 ± 0.01 | 0.16 ± 0.01 |  | 0.22 ± 0.01 | 0.17 ± 0.02 |
|  | 4.1 | 0.15 ± 0.02 | 0.17 ± 0.01 |  | 0.15 ± 0.05 | 0.16 ± 0.01 |
|  | 4.4 | 0.23 ± 0.01 | 0.16 ± 0.07 |  | 0.19 ± 0.02 | 0.21 ± 0.02 |
|  | **Grand mean** | 0.21 ± 0.03 | 0.17 ± 0.02 |  | 0.21 ± 0.03 | 0.17 ± 0.02 |
| **Drought** | 1.1 | 0.15 ± 0.04 | 0.12 ± 0.04 |  | 0.11 ± 0.04 | 0.12 ± 0.04 |
|  | 1.3 | 0.10 ± 0.03 | 0.10 ± 0.04 |  | 0.10 ± 0.04 | 0.11 ± 0.04 |
|  | 2.2 | 0.16 ± 0.05 | 0.12 ± 0.04 |  | 0.13 ± 0.04 | 0.13 ± 0.05 |
|  | 2.4 | 0.15 ± 0.04 | 0.13 ± 0.03 |  | 0.15 ± 0.04 | 0.12 ± 0.03 |
|  | 3.1 | 0.14 ± 0.04 | 0.07 ± 0.03 |  | 0.13 ± 0.04 | 0.11 ± 0.05 |
|  | 3.4 | 0.16 ± 0.02 | 0.11 ± 0.05 |  | 0.17 ± 0.04 | 0.11 ± 0.04 |
|  | 4.2 | 0.16 ± 0.04 | 0.11 ± 0.04 |  | 0.18 ± 0.05 | 0.16 ± 0.05 |
|  | 4.3 | 0.18 ± 0.04 | 0.12 ± 0.05 |  | 0.16 ± 0.06 | 0.12 ± 0.05 |
|  | **Grand mean** | 0.15 ± 0.02 | 0.11 ± 0.02 |  | 0.14 ± 0.03 | 0.12 ± 0.02 |

**Table S2.** Predawn leaf water potential (Mean ± SE, MPa) measured during the last month of the drought experiment (August) across a subset of 58 saplings (mesic population *Mastrils* and xeric population *Saxon*) on acidic soil.

| **Treatment** | **Chamber** | **2013** |  | **2014** |
| --- | --- | --- | --- | --- |
| **Control** | 1.2 | -0.15 ± 0.09 |  | -0.17 ± 0.06 |
|  | 1.4 | -0.18 ± 0.09 |  | -0.25 ± 0.13 |
|  | 2.1 | -0.16 ± 0.05 |  | -0.13 ± 0.05 |
|  | 2.3 | -0.16 ± 0.08 |  | -0.15 ± 0.07 |
|  | 3.2 | -0.18 ± 0.06 |  | -0.23 ± 0.05 |
|  | 3.3 | -0.18 ± 0.06 |  | -0.23 ± 0.13 |
|  | 4.1 | -0.11 ± 0.03 |  | -0.20 ± 0.08 |
|  | 4.4 | -0.55 ± 0.49 |  | -0.25 ± 0.07 |
|  | **Grand mean** | -0.21 ± 0.03 |  | -0.20 ± 0.02 |
| **Drought** | 1.1 | -1.28 ± 0.30 |  | -1.95 ± 0.17 |
|  | 1.3 | -1.17 ± 0.21 |  | -1.60 ± 0.10 |
|  | 2.2 | -1.53 ± 0.15 |  | -2.03 ± 0.05 |
|  | 2.4 | -1.33 ± 0.06 |  | -1.13 ± 0.29 |
|  | 3.1 | -1.60 ± 0.61 |  | -1.40 ± 0.61 |
|  | 3.4 | -1.25 ± 0.40 |  | -1.95 ± 0.17 |
|  | 4.2 | -1.80 ± 0.80 |  | -2.25 ± 0.25 |
|  | 4.3 | -1.23 ± 0.74 |  | -1.88 ± 0.10 |
|  | **Grand mean** | -1.40 ± 0.09 |  | -1.81 ± 0.07 |

**Table S3.** Variance components (a) and test of fixed effects (b) from the mixed-effects model for F_V_/F_M_^.^

**a**

| **Source** | **Variance** | **% of total** | **Standard error** | ***Z*** | ***P*** |
| --- | --- | --- | --- | --- | --- |
| Population | 0.000004 | 0.28% | 0.000023 | 0.183936 | 0.427 |
| Population*Treatment | 0.000000 | 0.00% | * | * | * |
| Population*Chamber | 0.000000 | 0.00% | * | * | * |
| Error | 0.001544 | 99.72% | 0.000118 | 13.117210 | 0.000 |
| Total | 0.001549 |  |  |  |  |

**b**

| **Term** | **Numerator df** | **Denominator df** | ***F*** | ***P*** |
| --- | --- | --- | --- | --- |
| Treatment | 1.00 | 345.58 | 1.03 | 0.312 |
| Chamber | 14.00 | 345.16 | 2.69 | 0.001 |

**Table S4.** Variance components (a) and test of fixed effects (b) from the mixed-effects model for PI_abs_^.^

**a**

| **Source** | **Var** | **% of total** | **Standard error** | ***Z*** | ***P*** |
| --- | --- | --- | --- | --- | --- |
| Population | 0.000000 | 0.00% | * | * | * |
| Population*Treatment | 0.000000 | 0.00% | * | * | * |
| Population*Chamber | 0.000000 | 0.00% | * | * | * |
| Error | 1.831681 | 100.00% | 0.137483 | 13.322913 | 0.000 |
| Total | 1.831681 |  |  |  |  |

**b**

| **Term** | **Numerator df** | **Denominator df** | ***F*** | ***P*** |
| --- | --- | --- | --- | --- |
| Treatment | 1.00 | 355.00 | 15.24 | 0.000 |
| Chamber | 14.00 | 355.00 | 7.79 | 0.000 |

**Table S5.** Variance components (a) and test of fixed effects (b) from the mixed-effects model for PI_tot_.

**a**

| **Source** | **Var** | **% of total** | **Standard error** | ***Z*** | ***P*** |
| --- | --- | --- | --- | --- | --- |
| Population | 0.010442 | 1.42% | 0.015731 | 0.663776 | 0.253 |
| Population*Treatment | 0.000000 | 0.00% | * | * | * |
| Population*Chamber | 0.074043 | 10.06% | 0.058512 | 1.265417 | 0.103 |
| Error | 0.651531 | 88.52% | 0.069032 | 9.438096 | 0.000 |
| Total | 0.736015 |  |  |  |  |

**b**

| **Term** | **Numerator df** | **Denominator df** | ***F*** | ***P*** |
| --- | --- | --- | --- | --- |
| Treatment | 1.00 | 155.80 | 33.69 | 0.000 |
| Chamber | 14.00 | 155.46 | 6.81 | 0.000 |

**Table S6.** Variance components (a) and test of fixed effects (b) from the Mixed Effects Model for SDG 2013.

**a**

| **Source** | **Var** | **% of total** | **Standard error** | ***Z*** | ***P*** |
| --- | --- | --- | --- | --- | --- |
| Population | 0.183440 | 4.34% | 0.116295 | 1.577365 | 0.057 |
| Population*treatment | 0.000000 | 0.00% | * | * | * |
| Population*soil | 0.000000 | 0.00% | * | * | * |
| Population*chamber | 0.182746 | 4.32% | 0.187518 | 0.974554 | 0.165 |
| Population*treatment*soil | 0.014325 | 0.34% | 0.079176 | 0.180932 | 0.428 |
| Population*chamber*soil | 0.451254 | 10.67% | 0.284260 | 1.587471 | 0.056 |
| Error | 3.397378 | 80.33% | 0.253336 | 13.410549 | 0.000 |
| Total | 4.229143 |  |  |  |  |

**b**

| **Term** | **Numerator df** | **Denominator df** | ***F*** | ***P*** |
| --- | --- | --- | --- | --- |
| Treatment | 1.00 | 32.53 | 18.88 | 0.000 |
| Soil | 1.00 | 30.07 | 0.68 | 0.417 |
| Chamber | 14.00 | 161.27 | 0.95 | 0.509 |
| Treatment*soil | 1.00 | 30.06 | 1.09 | 0.304 |
| Chamber*soil | 14.00 | 153.15 | 0.54 | 0.906 |

**Table S7.** Variance components (a) and test of fixed effects (b) from the Mixed Effects Model for SDG 2014.

**a**

| **Source** | **Var** | **% of total** | **Standard error** | ***Z*** | ***P*** |
| --- | --- | --- | --- | --- | --- |
| Population | 0.137805 | 5.61% | 0.087959 | 1.566700 | 0.059 |
| Population*treatment | 0.000000 | 0.00% | * | * | * |
| Population*soil | 0.019178 | 0.78% | 0.054164 | 0.354083 | 0.362 |
| Population*chamber | 0.087716 | 3.57% | 0.088438 | 0.991833 | 0.161 |
| Population*treatment*soil | 0.022825 | 0.93% | 0.057426 | 0.397476 | 0.346 |
| Population*chamber*soil | 0.000000 | 0.00% | * | * | * |
| Error | 2.188056 | 89.11% | 0.144876 | 15.102916 | 0.000 |
| Total | 2.455580 |  |  |  |  |

**b**

| **Term** | **Numerator df** | **Denominator df** | ***F*** | ***P*** |
| --- | --- | --- | --- | --- |
| Treatment | 1.00 | 23.76 | 97.36 | 0.000 |
| Soil | 1.00 | 11.18 | 18.57 | 0.001 |
| Chamber | 14.00 | 153.07 | 1.14 | 0.328 |
| Treatment*soil | 1.00 | 19.49 | 3.49 | 0.067 |
| Chamber*soil | 14.00 | 479.79 | 0.69 | 0.782 |

**Table S8.** Variance components (a) and test of fixed effects (b) from the Mixed Effects Model for SDG 2013-2014.

**a**

| **Source** | **Var** | **% of total** | **Standard error** | ***Z*** | ***P*** |
| --- | --- | --- | --- | --- | --- |
| Population | 0.796680 | 8.60% | 0.435165 | 1.830753 | 0.034 |
| Population*treatment | 0.000000 | 0.00% | * | * | * |
| Population*soil | 0.009112 | 0.10% | 0.170612 | 0.053409 | 0.479 |
| Population*chamber | 0.702105 | 7.58% | 0.414693 | 1.693071 | 0.045 |
| Population*treatment*soil | 0.073169 | 0.79% | 0.212907 | 0.343667 | 0.366 |
| Population*chamber*soil | 0.264043 | 2.85% | 0.581070 | 0.454408 | 0.325 |
| Error | 7.413506 | 80.07% | 0.581517 | 12.748556 | 0.000 |
| Total | 9.258615 |  |  |  |  |

**b**

| **Term** | **Numerator df** | **Denominator df** | ***F*** | ***P*** |
| --- | --- | --- | --- | --- |
| Treatment | 1.00 | 26.93 | 58.46 | 0.000 |
| Soil | 1.00 | 11.37 | 4.11 | 0.067 |
| Chamber | 14.00 | 155.10 | 0.98 | 0.475 |
| Treatment*soil | 1.00 | 18.10 | 2.14 | 0.161 |
| Chamber*soil | 14.00 | 151.58 | 0.35 | 0.986 |

**Table S9.** Spearman’s rank correlation coefficients (*rho*) and their corresponding *P*-values between individual heterozygosity and traits under different experimental conditions and for all saplings pooled together.

| **Trait** | **Drought** | | **Drought/Acidic Soil** | | **Drought/Calcareous Soil** | | **Control** | | **Control/Acidic Soil** | | **Control/Calcareous Soil** | | **All Saplings** | |
| --- | --- | --- | --- | --- | --- | --- | --- | --- | --- | --- | --- | --- | --- | --- |
|  | ***rho*** | ***P*** | ***rho*** | ***P*** | ***rho*** | ***P*** | ***rho*** | ***P*** | ***rho*** | ***P*** | ***rho*** | ***P*** | ***rho*** | ***P*** |
| **F_V_/F_M_** | ND | ND | 0.16 | **0.03** | ND | ND | ND | ND | 0.00 | 0.98 | ND | ND | 0.09 | **0.01** |
| **PI_abs_** | ND | ND | 0.06 | 0.38 | ND | ND | ND | ND | -0.06 | 0.41 | ND | ND | -0.02 | 0.62 |
| **PI_tot_** | ND | ND | 0.07 | 0.32 | ND | ND | ND | ND | -0.11 | 0.13 | ND | ND | -0.02 | 0.58 |
| **SDG 2013** | 0.10 | **0.04** | 0.05 | 0.50 | 0.17 | **0.02** | -0.05 | 0.35 | -0.03 | 0.72 | -0.11 | 0.13 | 0.03 | 0.40 |
| **SDG 2014** | 0.07 | 0.16 | 0.04 | 0.58 | 0.13 | 0.09 | -0.07 | 0.16 | -0.03 | 0.71 | -0.13 | 0.10 | 0.00 | 0.98 |
| **SDG 2013-14** | 0.06 | 0.21 | 0.01 | 0.86 | 0.14 | 0.07 | -0.07 | 0.15 | -0.03 | 0.65 | -0.13 | 0.10 | 0.00 | 0.96 |

Significant *P*-values are highlighted by bold. ND - not determined

**Table S10.** Number of saplings from each population used under each treatment and soil in the drought experiment.

| **Population** | **Treatment** | **Soil** | **N** |
| --- | --- | --- | --- |
| Felsberg | Control | Acidic | 15 |
|  |  | Calcareous | 14 |
|  | Drought | Acidic | 17 |
|  |  | Calcareous | 15 |
| Chur | Control | Acidic | 15 |
|  |  | Calcareous | 16 |
|  | Drought | Acidic | 16 |
|  |  | Calcareous | 16 |
| Malans | Control | Acidic | 16 |
|  |  | Calcareous | 16 |
|  | Drought | Acidic | 16 |
|  |  | Calcareous | 16 |
| Mastrils | Control | Acidic | 16 |
|  |  | Calcareous | 16 |
|  | Drought | Acidic | 16 |
|  |  | Calcareous | 14 |
| Sargans | Control | Acidic | 15 |
|  |  | Calcareous | 16 |
|  | Drought | Acidic | 16 |
|  |  | Calcareous | 16 |
| Mels | Control | Acidic | 14 |
|  |  | Calcareous | 15 |
|  | Drought | Acidic | 16 |
|  |  | Calcareous | 15 |
| Ardon | Control | Acidic | 16 |
|  |  | Calcareous | 15 |
|  | Drought | Acidic | 16 |
|  |  | Calcareous | 16 |
| Chamoson | Control | Acidic | 16 |
|  |  | Calcareous | 16 |
|  | Drought | Acidic | 16 |
|  |  | Calcareous | 16 |
| Saxon | Control | Acidic | 16 |
|  |  | Calcareous | 16 |
|  | Drought | Acidic | 16 |
|  |  | Calcareous | 16 |
| Martigny | Control | Acidic | 16 |
|  |  | Calcareous | 16 |
|  | Drought | Acidic | 16 |
|  |  | Calcareous | 16 |
| Collombey | Control | Acidic | 16 |
|  |  | Calcareous | 16 |
|  | Drought | Acidic | 16 |
|  |  | Calcareous | 15 |
| Ollon | Control | Acidic | 16 |
|  |  | Calcareous | 15 |
|  | Drought | Acidic | 16 |
|  |  | Calcareous | 16 |
| Total | Control | Acidic | 188 |
|  |  | Calcareous | 187 |
|  | Drought | Acidic | 193 |
|  |  | Calcareous | 187 |
| **Grand total** | | | 755 |

**Table S11.** Summary on the number of saplings from each population under each treatment, type of soil and chamber.

| **Control** | | | | **Drought** | | | |
| --- | --- | --- | --- | --- | --- | --- | --- |
| **Chamber** | **Soil** | **Population** | **N** | **Chamber** | **Soil** | **Population** | **N** |
| ch1.2 | Acidic | Felsberg | 1 | ch1.1 | Acidic | Felsberg | 2 |
|  |  | Chur | 2 |  |  | Chur | 2 |
|  |  | Malans | 2 |  |  | Malans | 2 |
|  |  | Mastrils | 2 |  |  | Mastrils | 2 |
|  |  | Sargans | 2 |  |  | Sargans | 2 |
|  |  | Mels | 2 |  |  | Mels | 2 |
|  |  | Ardon | 2 |  |  | Ardon | 2 |
|  |  | Chamoson | 2 |  |  | Chamoson | 2 |
|  |  | Saxon | 2 |  |  | Saxon | 2 |
|  |  | Martigny | 2 |  |  | Martigny | 2 |
|  |  | Collombey | 2 |  |  | Collombey | 2 |
|  |  | Ollon | 2 |  |  | Ollon | 2 |
|  | Calcareous | Felsberg | 2 |  | Calcareous | Felsberg | 2 |
|  |  | Chur | 2 |  |  | Chur | 2 |
|  |  | Malans | 2 |  |  | Malans | 2 |
|  |  | Mastrils | 2 |  |  | Mastrils | 1 |
|  |  | Sargans | 2 |  |  | Sargans | 2 |
|  |  | Mels | 2 |  |  | Mels | 2 |
|  |  | Ardon | 2 |  |  | Ardon | 2 |
|  |  | Chamoson | 2 |  |  | Chamoson | 2 |
|  |  | Saxon | 2 |  |  | Saxon | 2 |
|  |  | Martigny | 2 |  |  | Martigny | 2 |
|  |  | Collombey | 2 |  |  | Collombey | 2 |
|  |  | Ollon | 2 |  |  | Ollon | 2 |
| ch1.4 | Acidic | Felsberg | 2 | ch1.3 | Acidic | Felsberg | 2 |
|  |  | Chur | 2 |  |  | Chur | 2 |
|  |  | Malans | 2 |  |  | Malans | 2 |
|  |  | Mastrils | 2 |  |  | Mastrils | 2 |
|  |  | Sargans | 2 |  |  | Sargans | 2 |
|  |  | Mels | 1 |  |  | Mels | 2 |
|  |  | Ardon | 2 |  |  | Ardon | 2 |
|  |  | Chamoson | 2 |  |  | Chamoson | 2 |
|  |  | Saxon | 2 |  |  | Saxon | 2 |
|  |  | Martigny | 2 |  |  | Martigny | 2 |
|  |  | Collombey | 2 |  |  | Collombey | 2 |
|  |  | Ollon | 2 |  |  | Ollon | 2 |
|  | Calcareous | Felsberg | 2 |  | Calcareous | Felsberg | 2 |
|  |  | Chur | 2 |  |  | Chur | 2 |
|  |  | Malans | 2 |  |  | Malans | 2 |
|  |  | Mastrils | 2 |  |  | Mastrils | 2 |
|  |  | Sargans | 2 |  |  | Sargans | 2 |
|  |  | Mels | 2 |  |  | Mels | 2 |
|  |  | Ardon | 2 |  |  | Ardon | 2 |
|  |  | Chamoson | 2 |  |  | Chamoson | 2 |
|  |  | Saxon | 2 |  |  | Saxon | 2 |
|  |  | Martigny | 2 |  |  | Martigny | 2 |
|  |  | Collombey | 2 |  |  | Collombey | 2 |
|  |  | Ollon | 2 |  |  | Ollon | 2 |
| ch2.1 | Acidic | Felsberg | 2 | ch2.2 | Acidic | Felsberg | 2 |
|  |  | Chur | 2 |  |  | Chur | 2 |
|  |  | Malans | 2 |  |  | Malans | 2 |
|  |  | Mastrils | 2 |  |  | Mastrils | 2 |
|  |  | Sargans | 2 |  |  | Sargans | 2 |
|  |  | Mels | 2 |  |  | Mels | 2 |
|  |  | Ardon | 2 |  |  | Ardon | 2 |
|  |  | Chamoson | 2 |  |  | Chamoson | 2 |
|  |  | Saxon | 2 |  |  | Saxon | 2 |
|  |  | Martigny | 2 |  |  | Martigny | 2 |
|  |  | Collombey | 2 |  |  | Collombey | 2 |
|  |  | Ollon | 2 |  |  | Ollon | 2 |
|  | Calcareous | Felsberg | 1 |  | Calcareous | Felsberg | 2 |
|  |  | Chur | 2 |  |  | Chur | 2 |
|  |  | Malans | 2 |  |  | Malans | 2 |
|  |  | Mastrils | 2 |  |  | Mastrils | 2 |
|  |  | Sargans | 2 |  |  | Sargans | 2 |
|  |  | Mels | 2 |  |  | Mels | 2 |
|  |  | Ardon | 2 |  |  | Ardon | 2 |
|  |  | Chamoson | 2 |  |  | Chamoson | 2 |
|  |  | Saxon | 2 |  |  | Saxon | 2 |
|  |  | Martigny | 2 |  |  | Martigny | 2 |
|  |  | Collombey | 2 |  |  | Collombey | 2 |
|  |  | Ollon | 1 |  |  | Ollon | 2 |
| ch2.3 | Acidic | Felsberg | 1 | ch2.4 | Acidic | Felsberg | 2 |
|  |  | Chur | 2 |  |  | Chur | 2 |
|  |  | Malans | 2 |  |  | Malans | 2 |
|  |  | Mastrils | 2 |  |  | Mastrils | 2 |
|  |  | Sargans | 2 |  |  | Sargans | 2 |
|  |  | Mels | 2 |  |  | Mels | 0 |
|  |  | Ardon | 2 |  |  | Ardon | 2 |
|  |  | Chamoson | 2 |  |  | Chamoson | 2 |
|  |  | Saxon | 2 |  |  | Saxon | 2 |
|  |  | Martigny | 2 |  |  | Martigny | 2 |
|  |  | Collombey | 2 |  |  | Collombey | 2 |
|  |  | Ollon | 2 |  |  | Ollon | 2 |
|  | Calcareous | Felsberg | 2 |  | Calcareous | Felsberg | 2 |
|  |  | Chur | 2 |  |  | Chur | 2 |
|  |  | Malans | 2 |  |  | Malans | 2 |
|  |  | Mastrils | 2 |  |  | Mastrils | 2 |
|  |  | Sargans | 2 |  |  | Sargans | 2 |
|  |  | Mels | 2 |  |  | Mels | 2 |
|  |  | Ardon | 2 |  |  | Ardon | 2 |
|  |  | Chamoson | 2 |  |  | Chamoson | 2 |
|  |  | Saxon | 2 |  |  | Saxon | 2 |
|  |  | Martigny | 2 |  |  | Martigny | 2 |
|  |  | Collombey | 2 |  |  | Collombey | 2 |
|  |  | Ollon | 2 |  |  | Ollon | 2 |
| ch3.2 | Acidic | Felsberg | 2 | ch3.1 | Acidic | Felsberg | 2 |
|  |  | Chur | 2 |  |  | Chur | 2 |
|  |  | Malans | 2 |  |  | Malans | 2 |
|  |  | Mastrils | 2 |  |  | Mastrils | 2 |
|  |  | Sargans | 2 |  |  | Sargans | 2 |
|  |  | Mels | 1 |  |  | Mels | 2 |
|  |  | Ardon | 2 |  |  | Ardon | 2 |
|  |  | Chamoson | 2 |  |  | Chamoson | 2 |
|  |  | Saxon | 2 |  |  | Saxon | 2 |
|  |  | Martigny | 2 |  |  | Martigny | 2 |
|  |  | Collombey | 2 |  |  | Collombey | 2 |
|  |  | Ollon | 2 |  |  | Ollon | 2 |
|  | Calcareous | Felsberg | 1 |  | Calcareous | Felsberg | 2 |
|  |  | Chur | 2 |  |  | Chur | 2 |
|  |  | Malans | 2 |  |  | Malans | 2 |
|  |  | Mastrils | 2 |  |  | Mastrils | 2 |
|  |  | Sargans | 2 |  |  | Sargans | 2 |
|  |  | Mels | 2 |  |  | Mels | 2 |
|  |  | Ardon | 1 |  |  | Ardon | 2 |
|  |  | Chamoson | 2 |  |  | Chamoson | 2 |
|  |  | Saxon | 2 |  |  | Saxon | 2 |
|  |  | Martigny | 2 |  |  | Martigny | 2 |
|  |  | Collombey | 2 |  |  | Collombey | 2 |
|  |  | Ollon | 2 |  |  | Ollon | 2 |
| ch3.3 | Acidic | Felsberg | 2 | ch3.4 | Acidic | Felsberg | 2 |
|  |  | Chur | 2 |  |  | Chur | 2 |
|  |  | Malans | 2 |  |  | Malans | 2 |
|  |  | Mastrils | 2 |  |  | Mastrils | 2 |
|  |  | Sargans | 1 |  |  | Sargans | 2 |
|  |  | Mels | 2 |  |  | Mels | 2 |
|  |  | Ardon | 2 |  |  | Ardon | 2 |
|  |  | Chamoson | 2 |  |  | Chamoson | 2 |
|  |  | Saxon | 2 |  |  | Saxon | 2 |
|  |  | Martigny | 2 |  |  | Martigny | 2 |
|  |  | Collombey | 2 |  |  | Collombey | 2 |
|  |  | Ollon | 2 |  |  | Ollon | 2 |
|  | Calcareous | Felsberg | 2 |  | Calcareous | Felsberg | 2 |
|  |  | Chur | 2 |  |  | Chur | 2 |
|  |  | Malans | 2 |  |  | Malans | 2 |
|  |  | Mastrils | 2 |  |  | Mastrils | 2 |
|  |  | Sargans | 2 |  |  | Sargans | 2 |
|  |  | Mels | 1 |  |  | Mels | 2 |
|  |  | Ardon | 2 |  |  | Ardon | 2 |
|  |  | Chamoson | 2 |  |  | Chamoson | 2 |
|  |  | Saxon | 2 |  |  | Saxon | 2 |
|  |  | Martigny | 2 |  |  | Martigny | 2 |
|  |  | Collombey | 2 |  |  | Collombey | 2 |
|  |  | Ollon | 2 |  |  | Ollon | 2 |
| ch4.1 | Acidic | Felsberg | 2 | ch4.2 | Acidic | Felsberg | 2 |
|  |  | Chur | 1 |  |  | Chur | 2 |
|  |  | Malans | 2 |  |  | Malans | 2 |
|  |  | Mastrils | 2 |  |  | Mastrils | 2 |
|  |  | Sargans | 2 |  |  | Sargans | 2 |
|  |  | Mels | 2 |  |  | Mels | 2 |
|  |  | Ardon | 2 |  |  | Ardon | 2 |
|  |  | Chamoson | 2 |  |  | Chamoson | 2 |
|  |  | Saxon | 2 |  |  | Saxon | 2 |
|  |  | Martigny | 2 |  |  | Martigny | 2 |
|  |  | Collombey | 2 |  |  | Collombey | 2 |
|  |  | Ollon | 2 |  |  | Ollon | 2 |
|  | Calcareous | Felsberg | 2 |  | Calcareous | Felsberg | 2 |
|  |  | Chur | 2 |  |  | Chur | 2 |
|  |  | Malans | 2 |  |  | Malans | 2 |
|  |  | Mastrils | 2 |  |  | Mastrils | 1 |
|  |  | Sargans | 2 |  |  | Sargans | 2 |
|  |  | Mels | 2 |  |  | Mels | 1 |
|  |  | Ardon | 2 |  |  | Ardon | 2 |
|  |  | Chamoson | 2 |  |  | Chamoson | 2 |
|  |  | Saxon | 2 |  |  | Saxon | 2 |
|  |  | Martigny | 2 |  |  | Martigny | 2 |
|  |  | Collombey | 2 |  |  | Collombey | 1 |
|  |  | Ollon | 2 |  |  | Ollon | 2 |
| ch4.4 | Acidic | Felsberg | 3 | ch4.3 | Acidic | Felsberg | 3 |
|  |  | Chur | 2 |  |  | Chur | 2 |
|  |  | Malans | 2 |  |  | Malans | 2 |
|  |  | Mastrils | 2 |  |  | Mastrils | 2 |
|  |  | Sargans | 2 |  |  | Sargans | 2 |
|  |  | Mels | 2 |  |  | Mels | 2 |
|  |  | Ardon | 2 |  |  | Ardon | 2 |
|  |  | Chamoson | 2 |  |  | Chamoson | 2 |
|  |  | Saxon | 2 |  |  | Saxon | 2 |
|  |  | Martigny | 2 |  |  | Martigny | 2 |
|  |  | Collombey | 2 |  |  | Collombey | 2 |
|  |  | Ollon | 2 |  |  | Ollon | 2 |
|  | Calcareous | Felsberg | 2 |  | Calcareous | Felsberg | 1 |
|  |  | Chur | 2 |  |  | Chur | 2 |
|  |  | Malans | 2 |  |  | Malans | 2 |
|  |  | Mastrils | 2 |  |  | Mastrils | 2 |
|  |  | Sargans | 2 |  |  | Sargans | 2 |
|  |  | Mels | 2 |  |  | Mels | 2 |
|  |  | Ardon | 2 |  |  | Ardon | 2 |
|  |  | Chamoson | 2 |  |  | Chamoson | 2 |
|  |  | Saxon | 2 |  |  | Saxon | 2 |
|  |  | Martigny | 2 |  |  | Martigny | 2 |
|  |  | Collombey | 2 |  |  | Collombey | 2 |
|  |  | Ollon | 2 |  |  | Ollon | 2 |

**Table S12.** Twenty-three candidate genes and 70 polymorphic SNPs genotyped in the beech saplings*.

| **Gene (abbreviation)** | **SNP** | **Type** | **Alleles** | **GenBank or EMBL accession number** |
| --- | --- | --- | --- | --- |
| Seifert *et al.* 2012, Table 1 [63] | | | | |
| *Aldehyde dehydrogenase (ALDH)* | *ALDH_1* | NC | C/T | FR774766 |
|  | *ALDH_2* | NS | C/A |  |
|  | *ALDH_3* | NS | C/G |  |
|  | *ALDH_4* | S | T/A |  |
| *Isocitrate dehydrogenase (IDH)* | *IDH_1* | S | C/T | FR796392 (part 1) |
|  | *IDH_3* | NC | C/G | FR796392 (part 2) |
|  | *IDH_4* | S | G/A |  |
| *Ascorbate peroxidase (APX)* | *APX1_1* | S | C/T | FR774767 (part 1) |
|  | *APX4_1* | NC | G/T | FR775801 |
|  | *APX4_2* | NS | C/G |  |
| *Early responsive to dehydration (ERD)* | *ERD* | NC | G/A | FR775803 |
| *Dehydrin (DHN)* | *Dhn_1* | NS | C/G | FR772355 |
|  | *Dhn_2* | NS | C/G |  |
| *Glutathione peroxidase (GPX)* | *GPX* | NS | C/T | FR796394 |
| Müller *et al.* 2015a,b, Table 2 [65,66] | | | | |
| *Cysteine proteinase (CysPro)* | *CysPro_118* | S | C/G | LK022694 |
|  | *CysPro_202* | S | A/G |  |
|  | *CysPro_728* | NC | C/G |  |
|  | *CysPro_783* | NC | T/G |  |
| *Chloroplast chaperonin like (CP10)* | *CP10_65* | S | T/C | LK022689 |
|  | *CP10_67* | NS | T/C |  |
|  | *CP10_377* | NC | T/G |  |
|  | *CP10_442* | NC | C/G |  |
|  | *CP10_503* | S | C/G |  |
|  | *CP10_749* | S | C/G |  |
|  | *CP10_1317* | NC | A/G |  |
|  | *CP10_1428* | NS | T/C |  |
| *Dof zinc finger protein (DAG)* | *DAG_81* | NC | A/G | LK022690 |
|  | *DAG_289* | NC | A/T |  |
|  | *DAG_1059* | S | T/G |  |
| *Histone 3 (His3)* | *His3C1_292* | NC | T/C | LK022692 |
|  | *His3C2_104* | S | A/C | LK022693 |
|  | *His3C2_186* | NC | T/C |  |
|  | *His3C2_260* | S | A/G |  |
| *NAC transcription factor (NAC)* | *NAC_854* | NS | A/C | LK022695 |
|  | *NAC_962* | S | A/G |  |
|  | *NAC_1300* | NC | A/G |  |
| *Protein phosphatase 2C (PP2C)* | *PP2C_315* | NS | C/G | LK022696 |
|  | *PP2C_391* | S | T/G |  |
|  | *PP2C_791* | NS | A/G |  |
|  | *PP2C_941* | NC | T/G |  |
|  | *PP2C_1200* | S | A/G |  |
| Lalagüe *et al.* 2014, Appendix 1 [64] | | | | |
| *Xyloglucan endotransglucosylase / hydrolase 23 (XTH)* | *7_258* | NC | A/G | JX406438 |
|  | *7_520* | NC | T/G |  |
| *Short chain alcohol dehydrogenase (SDR)* | *17_880* | NC | T/C | JX406486 |
|  | *17_1081* | NC | T/G |  |
| *Potassium transporter 2 (KT2)* | *39_256* | S | A/C | JX406448 |
|  | *39_282* | NS | A/G |  |
| *CRT/DRE binding factor (DREB)* | *50_39* | NS | A/C | JX406449 |
|  | *50_232* | S | A/G |  |
| *S-adenosyl-l-homocysteine hydrolase (SAHH)* | *52_1_235* | NS | T/C | JX406452 |
|  | *52_1_368* | S | T/C |  |
| *Glyceraldehyde 3-phosphate dehydrogenase (GAPDH)* | *68_277* | NS | A/T | JX406456 |
|  | *68_313* | NC | A/C |  |
| *Light-harvesting complex II protein (LHCB2)* | *88_1_450* | NC | A/T | JX406459 |
|  | *88_1_727* | S | T/C |  |
|  | *88_1_803* | NS | C/G |  |
| *Catalase (CAT)* | *91_2_57* | S | T/C | JX406491 |
|  | *91_2_141* | S | A/C |  |
|  | *91_2_231* | S | T/G |  |
|  | *91_2_448* | NC | A/G |  |
|  | *91_2_479* | NC | T/C |  |
|  | *91_2_504* | NC | A/G |  |
| *1-aminocyclopropane-1-carboxylate oxidase (ACC-oxidase)* | *92_352* | NS | A/C | JX406462 |
|  | *92_630* | NC | T/C |  |
| *Cytosolic class I small heat-shock protein (sHsps)* | *110_1_293* | S | A/T | JX406466 |
|  | *110_1_423* | NS | T/G |  |
|  | *110_1_450* | NS | A/C |  |
| *Pectin methylesterase (PME)* | *154_2_137* | S | T/C | JX406481 |
|  | *154_2_371* | S | T/C |  |
|  | *154_2_617* | S | A/T |  |

***modified from Cuervo-Alarcon et al. (2018). *S* – synonymous, *NS* – non-synonymous, *NC* – non-coding; *APX1_1, APX4_1* and *APX4_2*, and *His3C1_292, His3C2_104, His3C2_186,* and *His3C2_260* represent the same genes *APX* and *His3*, respectively, but two different fragments for each with different accession numbers

**Table S13.** Assessment of normality for F_V_/F_M_ with Shapiro-Wilk test.

| **Population** | **Treatment** | **Shapiro-Wilk Statistic** | **df** | ***P*** |
| --- | --- | --- | --- | --- |
| Felsberg | Control | 0.691 | 15 | 0.000* |
|  | Drought | 0.686 | 17 | 0.000* |
| Chur | Control | 0.947 | 13 | 0.547 |
|  | Drought | 0.750 | 16 | 0.001* |
| Malans | Control | 0.940 | 15 | 0.382 |
|  | Drought | 0.479 | 16 | 0.000* |
| Mastrils | Control | 0.925 | 15 | 0.233 |
|  | Drought | 0.901 | 15 | 0.099 |
| Sargans | Control | 0.494 | 15 | 0.000* |
|  | Drought | 0.407 | 16 | 0.000* |
| Mels | Control | 0.858 | 13 | 0.036* |
|  | Drought | 0.727 | 16 | 0.000* |
| Ardon | Control | 0.530 | 16 | 0.000* |
|  | Drought | 0.587 | 16 | 0.000* |
| Chamoson | Control | 0.420 | 16 | 0.000* |
|  | Drought | 0.740 | 14 | 0.001* |
| Saxon | Control | 0.471 | 16 | 0.000* |
|  | Drought | 0.469 | 15 | 0.000* |
| Martigny | Control | 0.938 | 16 | 0.327 |
|  | Drought | 0.922 | 16 | 0.184 |
| Collombey | Control | 0.874 | 16 | 0.031* |
|  | Drought | 0.950 | 16 | 0.488 |
| Ollon | Control | 0.945 | 16 | 0.421 |
|  | Drought | 0.552 | 16 | 0.000* |

**Table S14.** Assessment of normality for PI_abs_ with Shapiro-Wilk test.

| **Population** | **Treatment** | **Shapiro-Wilk Statistic** | **df** | ***P*** |
| --- | --- | --- | --- | --- |
| Felsberg | Control | 0.750 | 15 | 0.001* |
|  | Drought | 0.917 | 17 | 0.132 |
| Chur | Control | 0.887 | 13 | 0.088 |
|  | Drought | 0.965 | 16 | 0.757 |
| Malans | Control | 0.853 | 15 | 0.019* |
|  | Drought | 0.976 | 16 | 0.923 |
| Mastrils | Control | 0.910 | 15 | 0.136 |
|  | Drought | 0.927 | 15 | 0.244 |
| Sargans | Control | 0.970 | 15 | 0.852 |
|  | Drought | 0.991 | 16 | 1.000 |
| Mels | Control | 0.879 | 13 | 0.070 |
|  | Drought | 0.966 | 16 | 0.764 |
| Ardon | Control | 0.886 | 16 | 0.048* |
|  | Drought | 0.971 | 16 | 0.855 |
| Chamoson | Control | 0.950 | 16 | 0.492 |
|  | Drought | 0.896 | 14 | 0.100 |
| Saxon | Control | 0.975 | 16 | 0.916 |
|  | Drought | 0.949 | 15 | 0.515 |
| Martigny | Control | 0.781 | 16 | 0.002* |
|  | Drought | 0.869 | 16 | 0.026* |
| Collombey | Control | 0.948 | 16 | 0.453 |
|  | Drought | 0.926 | 16 | 0.211 |
| Ollon | Control | 0.938 | 16 | 0.324 |
|  | Drought | 0.928 | 16 | 0.226 |

**Table S15.** Assessment of normality for PI_tot_ with Shapiro-Wilk test.

| **Population** | **Treatment** | **Shapiro-Wilk Statistic** | **df** | ***P*** |
| --- | --- | --- | --- | --- |
| Felsberg | Control | 0.914 | 15 | 0.157 |
|  | Drought | 0.952 | 17 | 0.482 |
| Chur | Control | 0.927 | 13 | 0.310 |
|  | Drought | 0.912 | 16 | 0.125 |
| Malans | Control | 0.922 | 15 | 0.207 |
|  | Drought | 0.946 | 16 | 0.427 |
| Mastrils | Control | 0.979 | 15 | 0.965 |
|  | Drought | 0.961 | 15 | 0.714 |
| Sargans | Control | 0.986 | 15 | 0.996 |
|  | Drought | 0.948 | 16 | 0.465 |
| Mels | Control | 0.958 | 13 | 0.718 |
|  | Drought | 0.948 | 16 | 0.465 |
| Ardon | Control | 0.978 | 16 | 0.948 |
|  | Drought | 0.947 | 16 | 0.451 |
| Chamoson | Control | 0.973 | 16 | 0.891 |
|  | Drought | 0.941 | 14 | 0.426 |
| Saxon | Control | 0.980 | 16 | 0.962 |
|  | Drought | 0.968 | 15 | 0.820 |
| Martigny | Control | 0.972 | 16 | 0.864 |
|  | Drought | 0.970 | 16 | 0.841 |
| Collombey | Control | 0.957 | 16 | 0.604 |
|  | Drought | 0.956 | 16 | 0.586 |
| Ollon | Control | 0.898 | 16 | 0.074 |
|  | Drought | 0.964 | 16 | 0.740 |

**Table S16.** Assessment of normality for SDG 2013 with Shapiro-Wilk test

| **Population** | **Treatment** | **Soil** | **Shapiro-Wilk Statistic** | **df** | ***P*** |
| --- | --- | --- | --- | --- | --- |
| Felsberg | Control | Acidic | 0.957 | 15 | 0.641 |
|  |  | Calcareous | 0.921 | 14 | 0.226 |
|  | Drought | Acidic | 0.914 | 17 | 0.118 |
|  |  | Calcareous | 0.970 | 15 | 0.853 |
| Chur | Control | Acidic | 0.965 | 15 | 0.786 |
|  |  | Calcareous | 0.921 | 16 | 0.175 |
|  | Drought | Acidic | 0.942 | 16 | 0.379 |
|  |  | Calcareous | 0.942 | 16 | 0.376 |
| Malans | Control | Acidic | 0.898 | 16 | 0.076 |
|  |  | Calcareous | 0.892 | 16 | 0.060 |
|  | Drought | Acidic | 0.818 | 16 | 0.005* |
|  |  | Calcareous | 0.889 | 15 | 0.064 |
| Mastrils | Control | Acidic | 0.873 | 16 | 0.030* |
|  |  | Calcareous | 0.879 | 16 | 0.037* |
|  | Drought | Acidic | 0.906 | 16 | 0.102 |
|  |  | Calcareous | 0.936 | 13 | 0.410 |
| Sargans | Control | Acidic | 0.980 | 15 | 0.971 |
|  |  | Calcareous | 0.948 | 15 | 0.491 |
|  | Drought | Acidic | 0.984 | 15 | 0.990 |
|  |  | Calcareous | 0.917 | 15 | 0.171 |
| Mels | Control | Acidic | 0.940 | 14 | 0.423 |
|  |  | Calcareous | 0.933 | 15 | 0.302 |
|  | Drought | Acidic | 0.959 | 14 | 0.699 |
|  |  | Calcareous | 0.955 | 14 | 0.649 |
| Ardon | Control | Acidic | 0.970 | 16 | 0.833 |
|  |  | Calcareous | 0.942 | 15 | 0.411 |
|  | Drought | Acidic | 0.942 | 15 | 0.402 |
|  |  | Calcareous | 0.887 | 16 | 0.051 |
| Chamoson | Control | Acidic | 0.986 | 16 | 0.994 |
|  |  | Calcareous | 0.895 | 16 | 0.067 |
|  | Drought | Acidic | 0.844 | 16 | 0.011 |
|  |  | Calcareous | 0.922 | 16 | 0.182 |
| Saxon | Control | Acidic | 0.804 | 15 | 0.004* |
|  |  | Calcareous | 0.911 | 16 | 0.120 |
|  | Drought | Acidic | 0.929 | 16 | 0.234 |
|  |  | Calcareous | 0.902 | 16 | 0.086 |
| Martigny | Control | Acidic | 0.962 | 16 | 0.694 |
|  |  | Calcareous | 0.966 | 16 | 0.765 |
|  | Drought | Acidic | 0.945 | 16 | 0.409 |
|  |  | Calcareous | 0.956 | 16 | 0.590 |
| Collombey | Control | Acidic | 0.949 | 16 | 0.473 |
|  |  | Calcareous | 0.913 | 15 | 0.153 |
|  | Drought | Acidic | 0.953 | 16 | 0.546 |
|  |  | Calcareous | 0.949 | 15 | 0.508 |
| Ollon | Control | Acidic | 0.934 | 16 | 0.285 |
|  |  | Calcareous | 0.950 | 14 | 0.554 |
|  | Drought | Acidic | 0.936 | 16 | 0.301 |
|  |  | Calcareous | 0.991 | 16 | 1.000 |

**Table S17.** Assessment of normality for SDG 2014 with Shapiro-Wilk test.

| **Population** | **Treatment** | **Soil** | **Shapiro-Wilk Statistic** | **df** | ***P*** |
| --- | --- | --- | --- | --- | --- |
| Felsberg | Control | Acidic | 0.936 | 13 | 0.410 |
|  |  | Calcareous | 0.901 | 12 | 0.165 |
|  | Drought | Acidic | 0.931 | 12 | 0.385 |
|  |  | Calcareous | 0.965 | 13 | 0.827 |
| Chur | Control | Acidic | 0.864 | 13 | 0.044* |
|  |  | Calcareous | 0.955 | 14 | 0.647 |
|  | Drought | Acidic | 0.983 | 16 | 0.985 |
|  |  | Calcareous | 0.956 | 15 | 0.617 |
| Malans | Control | Acidic | 0.937 | 15 | 0.351 |
|  |  | Calcareous | 0.917 | 14 | 0.200 |
|  | Drought | Acidic | 0.963 | 14 | 0.774 |
|  |  | Calcareous | 0.984 | 13 | 0.993 |
| Mastrils | Control | Acidic | 0.947 | 14 | 0.512 |
|  |  | Calcareous | 0.952 | 15 | 0.558 |
|  | Drought | Acidic | 0.884 | 15 | 0.055 |
|  |  | Calcareous | 0.965 | 12 | 0.852 |
| Sargans | Control | Acidic | 0.910 | 13 | 0.186 |
|  |  | Calcareous | 0.910 | 14 | 0.158 |
|  | Drought | Acidic | 0.959 | 14 | 0.709 |
|  |  | Calcareous | 0.874 | 14 | 0.047* |
| Mels | Control | Acidic | 0.972 | 13 | 0.920 |
|  |  | Calcareous | 0.950 | 15 | 0.520 |
|  | Drought | Acidic | 0.950 | 11 | 0.639 |
|  |  | Calcareous | 0.929 | 13 | 0.333 |
| Ardon | Control | Acidic | 0.885 | 14 | 0.068 |
|  |  | Calcareous | 0.860 | 15 | 0.024 |
|  | Drought | Acidic | 0.936 | 13 | 0.407 |
|  |  | Calcareous | 0.945 | 14 | 0.489 |
| Chamoson | Control | Acidic | 0.992 | 15 | 1.000 |
|  |  | Calcareous | 0.944 | 14 | 0.470 |
|  | Drought | Acidic | 0.945 | 11 | 0.586 |
|  |  | Calcareous | 0.956 | 15 | 0.617 |
| Saxon | Control | Acidic | 0.962 | 14 | 0.761 |
|  |  | Calcareous | 0.950 | 15 | 0.520 |
|  | Drought | Acidic | 0.959 | 14 | 0.703 |
|  |  | Calcareous | 0.905 | 16 | 0.096 |
| Martigny | Control | Acidic | 0.968 | 16 | 0.799 |
|  |  | Calcareous | 0.899 | 16 | 0.077 |
|  | Drought | Acidic | 0.828 | 16 | 0.007* |
|  |  | Calcareous | 0.959 | 16 | 0.650 |
| Collombey | Control | Acidic | 0.922 | 15 | 0.208 |
|  |  | Calcareous | 0.907 | 13 | 0.166 |
|  | Drought | Acidic | 0.964 | 16 | 0.726 |
|  |  | Calcareous | 0.965 | 15 | 0.785 |
| Ollon | Control | Acidic | 0.926 | 15 | 0.234 |
|  |  | Calcareous | 0.974 | 14 | 0.925 |
|  | Drought | Acidic | 0.953 | 14 | 0.609 |
|  |  | Calcareous | 0.974 | 16 | 0.894 |

**Table S18.** Assessment of normality for SDG 2013-2014 with Shapiro-Wilk test.

| **Population** | **Treatment** | **Soil** | **Shapiro-Wilk Statistic** | **df** | ***P*** |
| --- | --- | --- | --- | --- | --- |
| Felsberg | Control | Acidic | 0.981 | 14 | 0.982 |
|  |  | Calcareous | 0.936 | 12 | 0.453 |
|  | Drought | Acidic | 0.926 | 12 | 0.340 |
|  |  | Calcareous | 0.960 | 13 | 0.760 |
| Chur | Control | Acidic | 0.912 | 13 | 0.198 |
|  |  | Calcareous | 0.985 | 14 | 0.993 |
|  | Drought | Acidic | 0.933 | 16 | 0.269 |
|  |  | Calcareous | 0.937 | 15 | 0.343 |
| Malans | Control | Acidic | 0.932 | 15 | 0.291 |
|  |  | Calcareous | 0.921 | 14 | 0.230 |
|  | Drought | Acidic | 0.980 | 15 | 0.966 |
|  |  | Calcareous | 0.943 | 14 | 0.459 |
| Mastrils | Control | Acidic | 0.946 | 14 | 0.503 |
|  |  | Calcareous | 0.928 | 15 | 0.255 |
|  | Drought | Acidic | 0.930 | 15 | 0.270 |
|  |  | Calcareous | 0.977 | 12 | 0.966 |
| Sargans | Control | Acidic | 0.958 | 13 | 0.726 |
|  |  | Calcareous | 0.899 | 14 | 0.111 |
|  | Drought | Acidic | 0.977 | 15 | 0.945 |
|  |  | Calcareous | 0.917 | 14 | 0.197 |
| Mels | Control | Acidic | 0.935 | 13 | 0.395 |
|  |  | Calcareous | 0.965 | 15 | 0.783 |
|  | Drought | Acidic | 0.912 | 13 | 0.193 |
|  |  | Calcareous | 0.928 | 14 | 0.290 |
| Ardon | Control | Acidic | 0.949 | 14 | 0.549 |
|  |  | Calcareous | 0.961 | 15 | 0.710 |
|  | Drought | Acidic | 0.943 | 13 | 0.495 |
|  |  | Calcareous | 0.925 | 14 | 0.263 |
| Chamoson | Control | Acidic | 0.960 | 15 | 0.696 |
|  |  | Calcareous | 0.952 | 14 | 0.595 |
|  | Drought | Acidic | 0.883 | 11 | 0.115 |
|  |  | Calcareous | 0.921 | 15 | 0.202 |
| Saxon | Control | Acidic | 0.903 | 15 | 0.105 |
|  |  | Calcareous | 0.961 | 15 | 0.708 |
|  | Drought | Acidic | 0.966 | 14 | 0.821 |
|  |  | Calcareous | 0.938 | 16 | 0.322 |
| Martigny | Control | Acidic | 0.946 | 16 | 0.431 |
|  |  | Calcareous | 0.960 | 16 | 0.663 |
|  | Drought | Acidic | 0.973 | 16 | 0.891 |
|  |  | Calcareous | 0.923 | 16 | 0.190 |
| Collombey | Control | Acidic | 0.975 | 15 | 0.925 |
|  |  | Calcareous | 0.913 | 13 | 0.204 |
|  | Drought | Acidic | 0.966 | 16 | 0.779 |
|  |  | Calcareous | 0.982 | 15 | 0.980 |
| Ollon | Control | Acidic | 0.960 | 15 | 0.700 |
|  |  | Calcareous | 0.981 | 15 | 0.979 |
|  | Drought | Acidic | 0.981 | 15 | 0.976 |
|  |  | Calcareous | 0.980 | 16 | 0.962 |

**Table S19.** Assessment of homogeneity of variances for chlorophyll fluorescence traits by Levene’s test based on medians.

| **Trait** | **Levene Statistic** | **df1** | **df2** | ***P*** |
| --- | --- | --- | --- | --- |
| Fv/Fm | 0.565 | 23 | 347 | 0.949 |
| PI_abs_ | 1.059 | 23 | 347 | 0.390 |
| PI_tot_ | 1.331 | 23 | 347 | 0.143 |

**Table S20.** Assessment of homogeneity of variances for SDG traits by Levene’s test based on medians.

| **Trait** | **Levene Statistic** | **df1** | **df2** | ***P*** |
| --- | --- | --- | --- | --- |
| SDG 2013 | 0.756 | 47 | 694 | 0.884 |
| SDG 2014 | 1.328 | 47 | 631 | 0.075 |
| SDG 2013-2014 | 0.971 | 47 | 641 | 0.529 |

**Figure S1.** Quantile-quantile plots of estimated –Log_10_*P*-values for the chlorophyll fluorescence traits in the association analysis under different experimental conditions for all saplings (**a,** **b**), saplings under drought/acidic (**c**, **d**), and control/acidic (**e**) soil conditions. The red dots and blue triangles represent the observed *P*-values in GLM and MLM, respectively; the black line represents the expected *P*-values.

**Figure S2.** Quantile-quantile plots of the –Log_10_*P*-values for the stem diameter growth (SDG) estimated in association analysis in different years and under different experimental conditions for saplings under drought/acidic (**a**, **b**) and drought/calcareous (**c**, **d**) soil conditions. The red dots and blue triangles represent the observed *P*-values in GLM and MLM, respectively; the black line represents the expected *P*-values.

**Figure S3.** Frequency distribution of the pairwise relatedness coefficients (*r*_QG_) for saplings collected underneath different (DAT) and the same (SAT) adult trees.
